# Hypothalamic neuronal outputs transmit sensorimotor signals at the onset of locomotor initiation

**DOI:** 10.1016/j.isci.2023.108328

**Published:** 2023-10-24

**Authors:** Ekaterina Martianova, Renata Sadretdinova, Alicia Pageau, Nikola Pausic, Tommy Doucet Gentiletti, Danahé Leblanc, Arturo Marroquin Rivera, Benoît Labonté, Christophe D. Proulx

**Affiliations:** 1 CERVO Brain Research Center, Department of Psychiatry and Neurosciences, Université Laval, Québec, QC, Canada

**Keywords:** Behavioral neuroscience, Molecular neuroscience, Sensory neuroscience

## Abstract

The lateral hypothalamus (LH) plays a critical role in sensory integration to organize behavior responses. However, how projection-defined LH neuronal outputs dynamically transmit sensorimotor signals to major downstream targets to organize behavior is unknown. Here, using multi-fiber photometry, we show that three major LH neuronal outputs projecting to the dorsal raphe nucleus (DRN), ventral tegmental area (VTA), and lateral habenula (LHb) exhibit significant coherent activity in mice engaging sensory-evoked or self-initiated motor responses. Increased activity at LH axon terminals precedes movement initiation during active coping responses and the activity of serotonin neurons and dopamine neurons. The optogenetic activation of LH axon terminals in either of the DRN, VTA, or LHb was sufficient to increase motor initiation but had different effects on passive avoidance and sucrose consumption. Our findings support the complementary role of three projection-defined LH neuronal outputs in the transmission of sensorimotor signals to major downstream regions at movement onset.

## Highlights

• Distinct LH neuronal populations target the DRN, VTA, and LHb
• LH neuronal outputs have coherent activity
• Activity at the LH neuronal outputs coincides with mobility onset
• Optogenetic activation of LH neuronal outputs increases motor activity

## Graphical abstract

**Figure:**
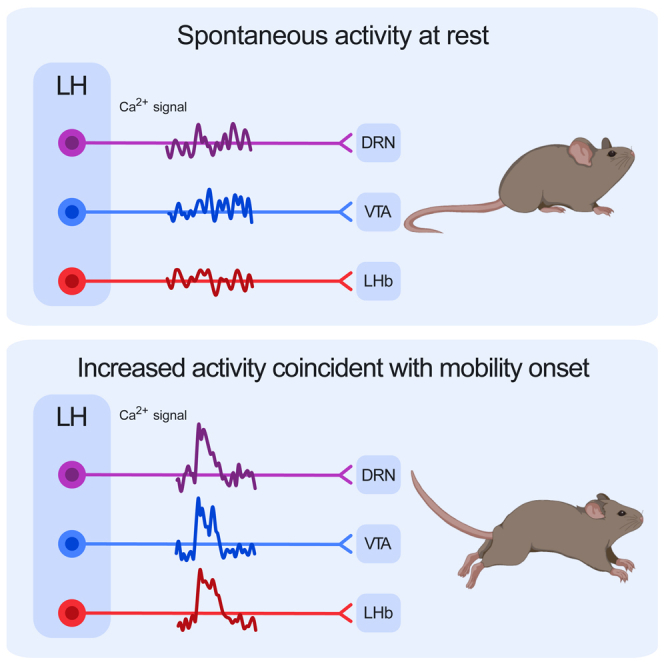

## Introduction

An animal that wishes to maximize its survival must select optimal behavioral responses in order to correctly adapt to its environment. Deciding to execute or refrain from a specific action depends largely on the actual state of the environment that needs to be accurately integrated and then translated into adaptive responses.^1^^,^^2^ In other words, an animal will engage active behavioral responses to obtain a reward, avoid a punishment, or escape from an aversive context. Alternatively, it can engage passive behaviors such as freezing and immobility to avoid being seen by a predator or to avoid exhaustion. Consequently, different cortical and subcortical brain regions have complementary roles to integrate context-dependent and sensory information to organize proper behavior responses.^3^

The lateral hypothalamus (LH) is a heterogeneous brain region that has been associated with a variety of behaviors related to motivation, reward, stress response, arousal, and feeding (reviewed by Bonnavion et al.^4^ and Fakhoury et al.^5^). More recently, it has been shown that activity of genetically defined LH neuronal populations in the LH is rapidly modulated by external sensory stimuli and during food consumption.^6^^,^^7^ LH activity also associates with movement, particularly during motor initiation when presented with sensory stimulation or self-initiated movement.^8^ These findings are suggesting that the LH plays an important role for sensory integration and motor control.^8^

The LH receives signals from many brain regions and sends neuronal efferents to major brain regions that have been associated with signal processing and sensorimotor control. It is then perfectly positioned to translate sensory signals into action. However, no studies have explored how multiple LH neuronal inputs or outputs are dynamically recruited in live animals performing behavioral tasks. Here, we thus aimed to examine signal transmission at three major projection-defined LH neuronal outputs during sensory-evoked or self-initiated behavior responses, namely the dorsal raphe nucleus (DRN), the ventral tegmental area (VTA), and the lateral habenula (LHb).

The DRN, main serotonin center, has long been implicated in a variety of sensorimotor, cognitive, and affective functions.^9^ The role of serotoninergic (5-HT, 5-hydroxytryptamine) DRN neurons in aversive processing, behavioral inhibition, and impulse control has been shown,^10^^,^^11^ but optogenetic direct activation of serotoninergic DRN neurons increases mobility in an aversive task.^12^ On the other hand, serotoninergic DRN neurons have also been shown to respond to rewards and reinforce behaviors.^13^ Such functional complexity of the serotonin system is likely to be resolved by functionally dissecting its neuronal inputs and outputs.^14^^,^^15^^,^^16^^,^^17^^,^^18^ The DRN receives profuse projections from the LH.^19^^,^^20^^,^^21^^,^^22^ However, the function of LH projections to the DRN in sensorimotor control in live behaving animals is unknown.

On the other hand, the VTA is a central brain region involved in reward processing. It receives inputs from the LH promoting behavioral activation^23^ and controlling compulsive sucrose seeking.^24^ The LH projections to the VTA (LH→VTA pathway) also play important roles in providing information on aversive outcomes^25^ controlling innate defensive behaviors.^26^

Finally, the LHb, also receiving major input from the LH, is an important nucleus of the reward circuit, providing negative value signals to the serotoninergic and dopaminergic systems.^27^ Recent reports have shown that projections from LH to the LHb (LH→LHb pathway) are involved in signaling aversive stimuli and cues predicting them to control innate defensive behaviors or reward-related behaviors,^28^^,^^29^^,^^30^ but also in regulating feeding.^31^

Given the functional heterogeneity of these targeted regions, we anticipated that activity at the LH→DRN, LH→VTA, and LH→LHb pathways would show key differences in sensory integration and in motor control. Unexpectedly, our results show that these LH neuronal outputs have coherent activity dynamics during sensory integration, but also at the onset of motor initiation. Particularly, activity along these pathways precedes movement onset in the tail suspension test (TST). Moreover, optogenetic stimulation of LH terminals in the DRN, VTA, and LHb increased motor activation, while it had a different effect on sucrose consumption, suggesting that these projection-defined LH outputs may have complementary roles in sensory integration gating sensorimotor responses.

## Results

### Projections to the DRN, VTA, and LHb are predominantly from distinct LH neuronal populations projecting to a unique downstream target

**Figure 1:**
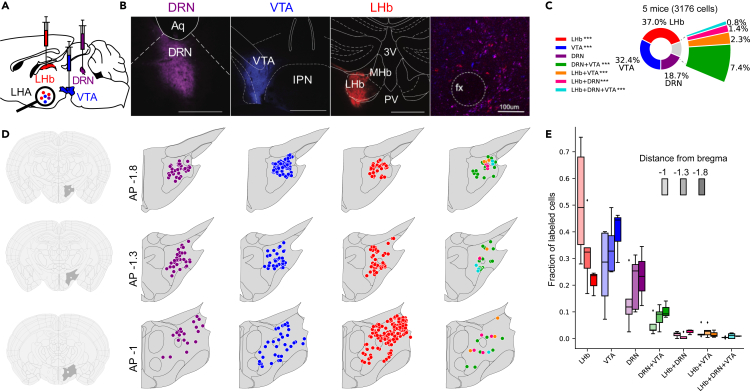
Projections to the DRN, VTA, and LHb are predominantly from distinct LH neuronal populations projecting to a unique downstream target (A) Schematic of the retrograde tracing strategy. (B) Examples of CTb injection in the DRN, VTA, and LHb, and representative confocal image showing LH neurons positive for CTb tracers. (C) Pie chart showing fraction of LH neurons projecting to DRN, VTA, LHb, or multiple projection sites. Chi-squared test assuming equal probability (1/7) of occurrence for each LH neuronal population. The p values were adjusted using the Bonferroni multiple testing correction method. ∗∗∗p < 0.001 for populations occurring significantly more or less frequently than expected. (D) Representative distribution of LH neurons, from three AP positions, classified as projecting to the DRN, VTA or LHb, or LH neurons projecting to two or three targets. (E) Fraction of LH projecting neurons classified as their AP position in the LH. Two-way ANOVA within factors tests (AP position) and pathways (LH→DRN, LH→VTA, and LH→LHb) with *post hoc* Tukey test. PV, paraventricular thalamus; 3V, third ventricle; Aq, aqueduct; IPN, interpeduncular nucleus; fx, fornix.

We first determined whether LH projections to the DRN, VTA, and LHb originate from overlapping or distinct neuronal populations. Mice were injected in the DRN, VTA, and LHb with the retrograde tracer cholera toxin subunit b (CTb), conjugated with different fluorophore, and CTb-containing LH neurons were examined (Figures 1A and 1B). We found that a large proportion of LH neurons project uniquely to either of the DRN, VTA or the LHb, while smaller fractions of LH neurons were positive for two or three tracers (Figure 1C). When comparing the expected frequencies of each of the LH populations through chi-squared test, assuming all the populations had the same probability of occurrence, we found that LH populations projecting uniquely to the LHb or the VTA were observed more frequently than what would be expected by chance while DRN-projecting LH neurons occurred as expected. However, LH projections targeting two or three downstream targets were all observed less frequently than what would be expected by chance. In other words, signal transmission from the LH is predominantly from distinct LH neuronal populations projecting to a single downstream target (DRN, VTA, or LHb). We also analyzed their distribution in the anterior-posterior axis in the LH (Figures 1D–1E). We did not observe significant difference in the distribution of the LH neurons labeled with CTb in the anterior-posterior axis. However, we observed that LHb-projecting LH neurons were more localized in the anterior LH while both DRN- and VTA-projecting LH were more localized in the posterior LH, which is consistent with previous findings.^32^

### The LH→DRN, LH→VTA, and LH→LHb pathways are activated by airpuffs and inhibited during sucrose consumption

**Figure 2:**
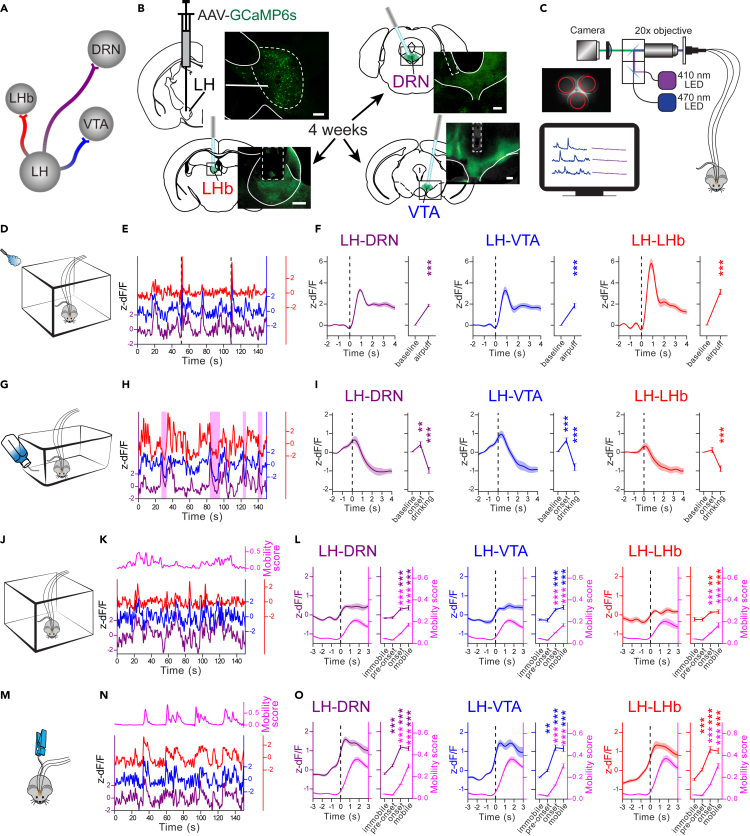
Dynamics at the LH→DRN, LH→VTA, and LH→LHb pathways to somatosensory stimulation and during sucrose consumption (A) Diagram of the LH neuronal outputs targeted in this work. (B) Diagram of the experimental setup and fluorescence images of GCaMP6s expression in LH neurons and axon terminals at the DRN, VTA, and LHb (scale bar 200 μm). (C) Diagram of the multi-fiber photometry calcium (Ca^2+^) imaging setup. (D) Experimental setup for the airpuffs. (E) Representative Ca^2+^ signal traces associated with the airpuffs (dashed vertical bars) simultaneously measured at the LH→DRN, LH→VTA, and LH→LHb pathways. (F) Peri-event plot of the average Ca^2+^ signal traces with all airpuff events at the LH→DRN (n = 12), LH→VTA (n = 11), and LH→LHb (n = 10) axon terminals. Plot of area under the curve (AUC) before and after the airpuffs. The lines represent means ± SEM (standard error of mean). Same convention as with D–F for the sucrose consumption test (G–I; LH→DRN (n = 13), LH→VTA (n = 12), LH→LHb (n = 12)), the open field test (J–L; LH→DRN (n = 11), LH→VTA (n = 7), LH→LHb (n = 5)), and the tail suspension test (M–O; LH→DRN (n = 8), LH→VTA (n = 6), LH→LHb (n = 6)). Sucrose consumption events are represented by pink shaded box in H. The magenta lines are mobility scores. Repeated measures three-way ANOVA between factors group (GCaMP6s- and eYFP-expressing mice) and within factors pathway (LH→DRN, LH→VTA, and LH→LHb), and time period (different for each test) with *post hoc* Dunnett’s test. The p values were adjusted using the Bonferroni multiple testing correction method. ∗p < 0.05, ∗∗p < 0.01, ∗∗∗p < 0.001. See also Figures S1–S5.

Next, to simultaneously examine activity at the LH→DRN, LH→VTA, and LH→LHb pathways, we injected an adeno-associated virus (AAV) encoding the calcium indicator GCaMP6s (AAV-CAG-GCaMP6s) in the LH of wild-type mice (Figure 2A). Optical fibers were implanted over the DRN, VTA, and LHb, allowing us to monitor activity at LH axon terminals projecting to the DRN, VTA, and LHb, respectively. Four weeks post-injection, GCaMP6s expression was detected in the cell bodies of the LH and in the axon terminals of these fibers in the DRN, VTA, and LHb (Figure 2B). Calcium-dependent change in fluorescence at axon terminals, which is a proxy for neuronal activity, was recorded simultaneously in all three pathways using multi-fiber photometry in freely behaving mice (Figure 2C).^33^ We first examined neuronal dynamics along these three projection-defined LH neuronal outputs coincident with the delivery of a brief somatosensory stimulus (airpuffs) or during consumption of a reward (2% sucrose solution).^6^^,^^34^ The mice were initially placed in an open-field arena, and 1-s airpuffs were delivered on the back of the animals at around 30 cm distance every 60 s (Figure 2D). Figure 2E shows representative traces where spontaneous activity was detected at all three pathways, which significantly increased coincidentally with the delivery of the airpuffs (APs) (Figure 2F). We then investigated how these pathways are modulated in an appetitive context, when mice consumed a reward. The same mice were water-deprived for 24 h and were then given free access to a 2% sucrose solution for 10 min (Figure 2G). In these sessions of sucrose consumption (SCT), the mice readily learned to drink from the sucrose dispenser. Drinking events were automatically detected with a lickometer and are represented by pink boxes in the representative traces shown in Figure 2H. Aligning the signals with the onset of drinking events revealed that activity at the LH→DRN and LH→VTA pathways increased significantly immediately prior to the onset of sucrose consumption. This change was not seen with the LH→LHb pathway. In all three pathways, however, activity significantly decreased during sucrose consumption (Figure 2I). Control mice injected with an AAV-eYFP exhibited no significant changes in fluorescence following the delivery of airpuffs (Figure S1C) or during sucrose consumption (Figure S1F), indicating that changes in fluorescence are dependent on changes in calcium and GCaMP6s signals and do not result from movement artifacts.

These results prompted us to examine whether sensorimotor signals were encoded at these pathways to control escape response to an airpuff or to motivate an approach to the reward port. We then repeated these experiments with some mice while tracking their movements. We observed a coincident increase in mobility (magenta line) at the delivery of APs (Figure S2A). Of note, significant activity remained high when mice returned to an immobility state (Figure S2A). No correlation was observed between neuronal activity and movement at the onset of sucrose consumption, but both activity and movement were inhibited during consumption, which significantly increased at the offset of drinking events (Figure S2B). Together, these results suggest that sensorimotor signals are encoded at the LH→DRN, LH→VTA, and LH→LHb pathways to control behavior responses.

### Coherent increase in activity at the LH→DRN, LH→VTA, and LH→LHb pathways coincides with the onset of mobility

To examine further the role of LH neuronal outputs during movement, activity was measured at LH neuronal outputs and movements were tracked from the mice previously used in the AP and SCT, but that were now free to explore either an open field (OFT) (Figures 2J–2L) or were suspended by the tail (TST) (Figures 2M–2O). OFT and TST are used to evaluate mobility (OFT) and motivated responses (TST),^16^^,^^35^ in which mice will engage periods of immobility and mobility. Figures 2K and 2N show representative activity traces monitored at the LH neuronal outputs and superimposed on the mobility score (magenta line), of a mouse placed in the OFT or a mouse suspended by its tail, respectively. In these contexts, activity at the LH→DRN, LH→VTA, and LH→LHb pathways significantly increased at mobility onset. We also observed a decrease in activity coincident with the offset of mobility (Figures S2C–S2D). In addition, mice placed in the TST exhibited a significant increase in activity at all three LH neuronal outputs immediately prior to the onset of mobility. Pearson correlation analysis between the activity of each of the pathways confirmed the coherence between these pathways, which we did not observe in eYFP-expressing mice (Figure S3). Taken together, these results show that these three major LH neuronal outputs exhibit coherent activity, which coincides with the onset and offset of mobility in the OFT and TST.

### Increased activity at the LH→DRN, LH→VTA, and LH→LHb pathways precedes mobility onset in the TST

**Figure 3:**
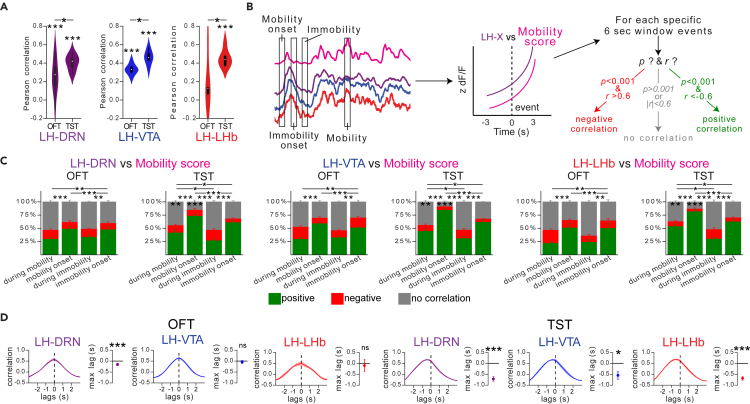
There is a high correlation between activity and mobility score at the LH→DRN, LH→VTA, and LH→LHb pathways during the TST (A) Pearson correlation between Ca^2+^ signal at the LH→DRN, LH→VTA, and LH→LHb pathways, and mobility scores during the OFT (LH→DRN (n = 11), LH→VTA (n = 7), LH→LHb (n = 4)) or TST (LH→DRN (n = 8), LH→VTA (n = 6), LH→LHb (n = 6)). One sample t test. Three-way mixed ANOVA between factors group (GCaMP6s- and eYFP-expressing mice), and within factors test (OFT and TST) and pathways (LH→DRN, LH→VTA, and LH→LHb) with *post hoc* Tukey test. The p values were adjusted using the Bonferroni multiple testing correction method. (B) Schematic of the event selection. Events at the onset mobility and immobility, and random events during mobility and immobility were chosen, and the Pearson correlation at 6 s peri-events between the Ca^2+^ signal and the mobility score was calculated. Correlations with p < 0.001 and r > 0.6 were considered as positive, p < 0.001 and r < 0.6 as negative, and the others as uncorrelated. (C) Fraction of positive (green), negative (red), and uncorrelated events (gray) in the OFT and TST for the LH→DRN, LH→VTA, and LH→LHb pathways. Four-way MANOVA between the factors group (GCaMP6s- and eYFP-expressing mice), test (OFT and TST), and pathways (LH→DRN, LH→VTA, and LH→LHb), and within factor events (during mobility, mobility onset, during immobility, immobility onset) for positive and negative correlations with a *post hoc* four-way mixed ANOVA and Tukey test for multiple comparisons. The p values were adjusted using the Bonferroni multiple testing correction method. (D) Cross-correlation analysis between the Ca^2+^ signal and the mobility score at mobility onset peri-events in the OFT (LH→DRN (n = 11), LH→VTA (n = 7), LH→LHb (n = 4)) and TST (LH→DRN (n = 8), LH→VTA (n = 6), LH→LHb (n = 6)). The lines represent means ± SEM of correlations vs. lag times and means ± SEM of lag times with maximum correlation. One sample t test. Two-way ANOVA within factors tests (OFT and TST) and pathways (LH→DRN, LH→VTA, and LH→LHb) with *post hoc* Tukey test. The p values were adjusted using the Bonferroni multiple testing correction method. ∗p < 0.05, ∗∗p < 0.01, ∗∗∗p < 0.001, ns - p > 0.1. See also Figure S6.

Pearson correlation analysis between LH neuronal output activity and mobility score measured with the OFT and TST showed a significant positive correlation, with the exception of the LH→LHb pathway with the OFT (Figure 3A), indicating that these pathways may play a role in transmitting signals controlling movement initiation. This correlation was significantly higher with mice tested in the TST, an aversive context characteristically engaging periods of mobility and immobility defined as active and passive coping strategies.^36^ To further investigate whether the high correlation between activity and the mobility score could be attributed to specific time events during the OFT and TST, we performed peri-event correlation analyses at specific time points (events). Events of four types were chosen: at mobility onset, at immobility onset, and at random time points during mobility and immobility. For each event, a Pearson correlation was measured for each 6-s peri-event traces of the mobility score and the calcium signal recorded at each of the LH outputs. Correlations with p < 0.001 and r > 0.6 were considered as positive correlation events, p < 0.001 and r < −0.6 as negative correlation events, and the others events as having no correlation. The number of positive, negative, and no correlations events were counted for each mouse, each time event, and each behavior test. The means ± SEM for all of the animals are given in Figure 3C. The statistical analysis revealed that the main difference between the groups was the number of positive correlations. The results were independent of the output (no difference by this factor or interactions with others). In other words, the number of positive correlations events was similar for the LH→DRN, LH→VTA, and LH→LHb pathways. This analysis revealed that the number of events showing positive correlation between activity and mobility was significantly higher at mobility onset and during mobility in the TST than in the OFT. The highest number of positive correlations events was observed at mobility onset during the TST. A cross-correlation analysis between activity and mobility score also confirmed that changes in activity from all three LH neuronal outputs preceded the change in mobility at mobility onset in the TST (Figure 3D). These results indicate that there is a significant correlation in activity between these three LH neuronal outputs and suggest that activity at these LH neuronal outputs may play an important role to initiate motor responses, particularly to engage an active coping response in a stressful context such as in the TST.^36^

### Ca^2+^ imaging of DRN- and VTA-projecting neurons in the LH also reveals coherent activity at these two projection-defined LH neuronal populations

**Figure 4:**
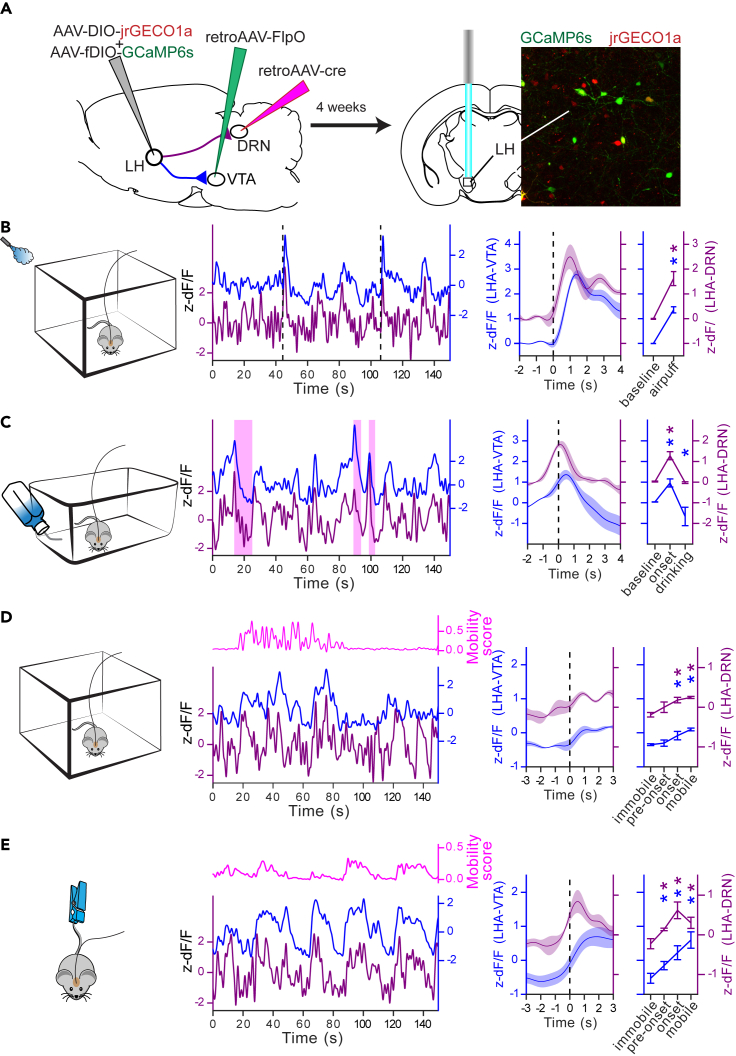
Ca^2+^ imaging from the LH neurons projecting to the DRN and the VTA (A) Diagram of the experimental setup and representative fluorescent image of LH neuron expressing GCaMP6s (green) and jrGECO1a (red) projecting to the VTA and DRN, respectively. (B) Representative Ca^2+^ signal traces associated with the airpuffs (dashed vertical bars) simultaneously measured from the LH neurons expressing GCaMP6s (projecting to the VTA) and jrGECO1a (projecting to the DRN) (left). Peri-event plot of the average Ca^2+^ signals to all the airpuff events at the LH→DRN and LH→VTA LH neurons, and plot of the average responses before and after the airpuffs (right) (n = 4). Lines represent the means ± SEM. Same convention as for B for the sucrose consumption test (C) (n = 3), the open field test (D) (n = 3), and the tail suspension test (E) (n = 3). The sucrose consumption events are represented by the pink shaded boxes in C. The magenta lines are the mobility scores. Repeated measures two-way ANOVA within factors pathway (LH→DRN and LH→VTA) and time periods (different for each test) with *post hoc* Dunnett’s test. The p values were adjusted using the Bonferroni multiple testing correction method. ∗p < 0.05. See also Figure S7.

Our CTb tracing experiment has shown that a small fraction of LH neurons send projections to both the VTA and the DRN (Figure 1C). Moreover, it is also possible that some signal measured at the VTA may overlap with signal transmission to the DRN. Consequently, to confirm that coherent activity measured in the VTA and DRN was from projection-defined LH neuronal outputs synapsing onto local neurons, and not from passing fibers, we used an intersectional viral approach to express the red-shifted calcium sensor jrGECO1a^37^ and GCaMP6s in DRN- and VTA-projecting LH neurons, respectively (Figure 4A).^33^^,^^38^ To this end, AAV vectors with retrograde properties,^39^ encoding the cre or FlpO recombinase, were injected into the DRN and the VTA, respectively. A cre-dependent AAV encoding jrGECO1a (AAV-DIO-jrGECO1a) and a FlpO-dependent AAV encoding GCaMP6s (AAV-fDIO-GCaMP6s) were injected into the LH of the same mice. This approach largely labeled non-overlapping LH neuronal populations (Figure 4A). An optical fiber was implanted over the LH of these mice to monitor signals from jrGECO1a and GCaMP6s expressed in DRN- and VTA-projecting LH neurons by dual-color fiber photometry.^33^ When the mice were tested in the same tasks described earlier, the calcium signals replicated the results previously obtained from the axon terminals recordings (Figures 4B–4E). These results support the fact that coherent signals we measured from axon terminals in DRN and VTA are from projection-defined LH neuronal outputs providing coherent signals to the DRN and VTA, which coincide with mobility onset.

### Activity at the LH→DRN, LH→VTA, and LH→LHb pathways increases at movement onset in an active avoidance task

**Figure 5:**
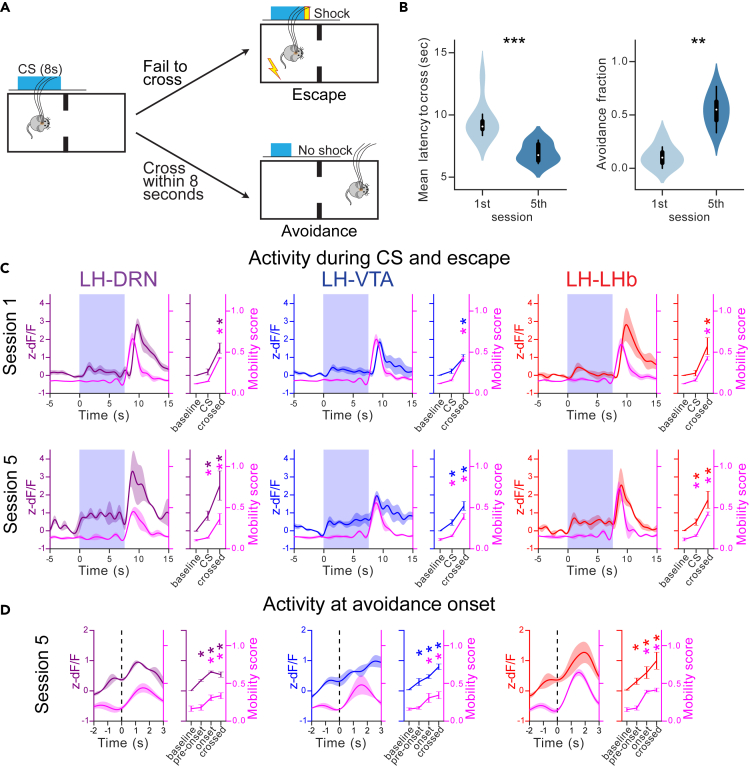
Activity at the LH→DRN, LH→VTA, and LH→LHb pathways increases at movement onset in an active avoidance task (A) Diagram of the avoidance conditioning test. The mice learn to shuttle within 8 s of conditioned stimuli (CS, tone and light) in order to avoid a foot shock. (B) The difference between early and late training trials is represented by the latency in shuttling from one compartment of the box to the other and the fraction of avoidance. Wilcoxon test: (B left): 6–8 mice per group, p value = 0.002335, (B right): 6–8 mice per group, p value = 0.000666. (C) Peri-event plots of average Ca^2+^ signals to all CS events followed by escape at the LH→DRN (n = 3), LH→VTA (n = 3), and LH→LHb (n = 6) axonal terminals and plots for AUC at baseline, during CS and escape. The lines represent means ± SEM. The top plots represent the results during early training, and the bottom plots represent the results during late training. (D) Same convention as C for mobility onsets of avoidance during late training. The magenta lines are mobility scores. Two-way repeated measures ANOVA within factors pathways (LH→DRN, LH→VTA, and LH→LHb) and time periods (different for CS and mobility onset) with *post hoc* Dunnett’s test. The p values were adjusted using the Bonferroni multiple testing correction method. ∗p < 0.05. See also Figure S8.

To further examine whether signals transmission at the projection-defined LH neuronal outputs correlate with motor initiation in mice engaging active coping responses, we trained a new group of mice in an active avoidance task while simultaneously monitoring activity at LH terminals in the DRN, VTA, and LHb. Previous works have shown that the LH→VTA and LH→LHb pathways encode aversive cues for active defensive behaviors,^25^^,^^26^^,^^30^ but the role of the LH→DRN pathway in these processes is unknown. In this task, the mice learned to associate a conditioned stimulus (CS) predicting an upcoming mild foot shock that could be escaped or avoided by crossing from one compartment to the other of the two-compartment box (Figure 5A). The mice quickly learned to avoid foot shocks, with the mean escape latency decreasing and the fraction of avoidance increasing with trained mice (session 5) (Figure 5B). Activity at the LH→DRN, LH→VTA, and LH→LHb pathways was monitored during the early (session 1) and late (session 5) stages of learning together with mobility scores (see STAR Methods). As previously shown, prediction signal of an upcoming mild foot shock developed at the LH→VTA, and LH→LHb pathways, but also at the LH→DRN pathway. These signals are independent of motor activation as seen in Figure 5C. Activity further increased at the onset of the foot shock which is coincident with the escape response during both session 1 and 5 (Figure 5C). We next specifically focused on avoidance events, in which mice engage motor response during the CS, shuttling to the other compartment of the two-compartment box, but before receiving the foot shock. We observed a significant increase in peri-event activity at onset of avoidance in all three pathways (Figure 5D). These results further support sensorimotor signal transmission in all three LH neuronal outputs at the onset of motor initiation (see Discussion).

### The LH provides monosynaptic excitatory and inhibitory inputs to serotoninergic and non-serotoninergic neurons in the DRN

**Figure 6:**
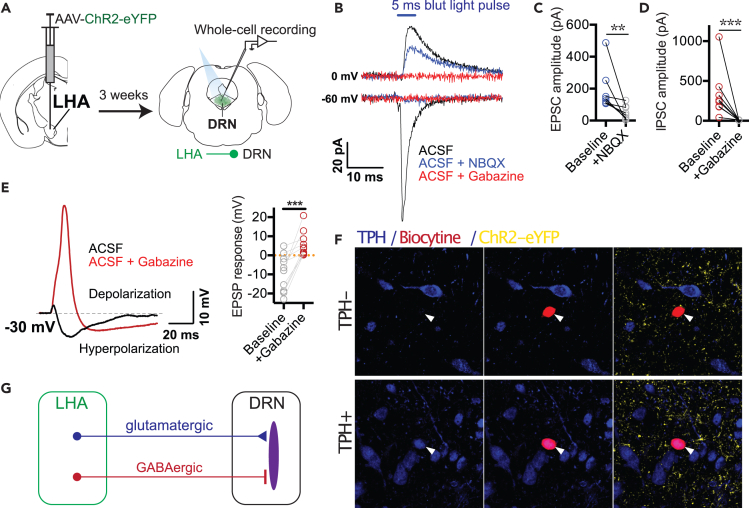
The LH provides monosynaptic excitatory and inhibitory projections to serotoninergic and non-serotoninergic neurons in the DRN (A) Diagram of the experimental setup. (B) Representative monosynaptic response from a DRN neuron following optogenetic stimulation of LH axon terminals. The response involves both an excitatory inward current measured at −60 mV and an inhibitory outward current measured at 0 mV, which were respectively blocked by the AMPA receptor antagonist (NBQX, blue) and the GABA-a receptor antagonist (gabazine, red). Plots of ChR2-evoked EPSCs (C) and IPSCs (D) (n = 8 cells) before and after blockade with the respective antagonists. (E) Representative monosynaptic response from a DRN neuron at a −30 mV holding voltage following optogenetic stimulation of LH axon terminals (left), and plots of EPSP amplitudes before and after GABA-a receptor blockade with gabazine (right) (n = 11 cells). Gabazine abolished the hyperpolarizing inhibitory component, resulting in large excitatory depolarizing responses. (F) Confocal images of DRN neurons filled with biocytin (red) during whole-cell patch-clamp recordings and *post hoc* immuno-labeling for the serotoninergic marker tryptophane hydroxylase (TPH, blue). LH axon terminals are shown in yellow in the DRN expressing ChR2-eYFP. (G) Schematic of connectivity between LH and DRN. Paired Wilcoxon test. ∗∗p < 0.01, ∗∗∗p < 0.001.

The LH neurons control the LHb through excitatory inputs^28^^,^^29^^,^^31^ while sending both excitatory and inhibitory projections to the VTA, differentially targeting dopaminergic and GABAergic neurons.^23^^,^^25^^,^^26^^,^^40^ To examine synaptic transmission from the LH to DRN, the LHs of wild-type mice were injected with an AAV encoding the excitatory opsin Channelrhodopsin-2 (ChR2) fused to the fluorescent protein eYFP (AAV-ChR2-eYFP) (Figure 6A). Three weeks later, acute brain slices encompassing the DRN were prepared, and whole-cell responses were obtained from DRN neurons (Figure 6A). All the recordings were made in the presence of TTX and 4-Aminopyridine (4-AP) to avoid polysynaptic responses. Recorded neurons were filled with biocytin and processed for *post hoc* staining for tryptophane hydroxylase (TPH) confirming that whole-cell recordings were from both serotoninergic or non-serotoninergic DRN neurons (Figure 6F). In voltage-clamp mode recordings, 5-ms light pulses evoked excitatory inward currents and inhibitory outward currents at −60 mV and 0 mV holding currents, respectively (Figure 6B). Monosynaptic excitatory and inhibitory transmissions were blocked with the AMPA receptor antagonist NBQX (Figure 6C) and the GABA-a receptor antagonist gabazine (Figure 6D), respectively. In whole-cell current clamp mode recordings with a −30 mV depolarized voltage and in presence of TTX and 4-AP, single light pulse induced a small depolarization followed by a large hyperpolarization (Figure 6E). In the presence of gabazine, however, the single light pulses revealed a large excitatory depolarizing component (Figure 6E). These results confirm that, similar to the VTA, LH sends convergent monosynaptic excitatory and inhibitory inputs to the DRN^21^ and suggest that LH inhibitory transmission gates excitatory transmission on both serotoninergic and non-serotoninergic neurons in the DRN (Figure 6G).

### Increased activity at the LH axon terminals precedes the increased activity of serotonin and dopamine neurons

**Figure 7:**
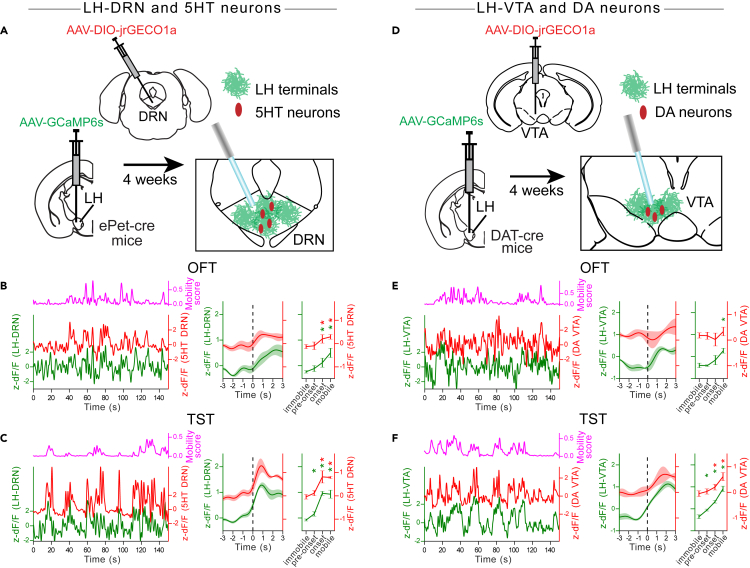
Increased activity of DRN^5HT^ and VTA^DA^ neurons follows increased activity in the LH→DRN and LH→VTA pathways at mobility onset in the TST (A and D) Diagrams of the experimental setups for simultaneous recordings from the DRN^5HT^ neurons and the LH→DRN pathway (A) and from the VTA^DA^ neurons and the LH→VTA pathway (D). (B and E) Representative Ca^2+^ signal traces from the DRN^5HT^ neurons and the LH→DRN pathway (B) (LH→DRN (n = 5), DRN^5HT^ (n = 5)) and the VTA^DA^ neurons and the LH→VTA pathway (E) (LH→VTA (n = 3), VTA^DA^ (n = 3)) during the OFT (left). Peri-event plots of the average Ca^2+^ signals at all mobility onsets and plots for AUC during immobility and mobility, and at mobility pre-onset and onset (right). The lines represent the means ± SEM (standard error of mean). Same convention as with B, E for the TST (C) (LH→DRN (n = 6), DRN^5HT^ (n = 4)), (F) (LH→VTA (n = 3), VTA^DA^ (n = 3)). The magenta lines are mobility scores. Repeated measures two-way ANOVA within factors pathways (DRN^5HT^ and LH→DRN, or VTA^DA^ and LH→VTA) and time periods (during immobility and mobility, at mobility pre-onset and onset) with post hoc Dunnett’s test. The p values were adjusted using the Bonferroni multiple testing correction method. ∗p < 0.05. See also Figures S9 and S10.

Since the LH provides both excitatory and inhibitory inputs to the DRN and VTA, we next wanted to determine whether the change in activity measured at LH axon terminals was correlated with a postsynaptic change in activity. To this end, we have performed dual-color fiber photometry recordings of LH axon terminals while co-monitoring the activity of serotonin and dopamine neurons in the DRN and VTA respectively. Specifically, a cre-dependent AAV encoding jrGECO1a (AAV-DIO-jrGECO1a) was injected into the DRN of ePet-cre mice, or in the VTA of DAT-ires-cre mice. These are transgenic mouse lines exclusively expressing the recombinase cre in 5-HT neurons^41^ and DA neurons,^42^ respectively. AAV-GCaMP6s was also injected into the LH of the same mice, and optical fibers were implanted in the DRN and the VTA as described earlier (Figures 7A and 7D). Four weeks post-injection, GCaMP6s expression was observed in the LH axons terminals and jrGECO1a expression was observed in the cell bodies of 5-HT neurons (Tph2+) and DA neurons (TH+) in the DRN and VTA, respectively (Figures S9A and S9D). This approach allowed us to simultaneously monitor activity in LH axons terminals (green fluorescence from GCaMP6s) and in genetically defined 5-HT and DA neurons (red fluorescence from jrGECO1a) using the same optical fiber. Representative traces show spontaneous activity from LH axon terminals as well as from DRN^5HT^ and VTA^DA^ neurons. The activity at DRN^5HT^ neurons significantly increased with somatosensory airpuff stimulation and decreased during sucrose consumption, replicating the results from the LH axon terminals (Figures S9B and S9E). However, the activity of VTA^DA^ neurons increased significantly with somatosensory airpuffs and during sucrose consumption (Figures S9C and S9F). When evaluated in the OFT and TST, the activity of VTA^DA^ neurons increased significantly at the onset of mobility in the TST, but not in the OFT (Figures 7B and 7C). However, the activity of DRN^5HT^ increased significantly at mobility onset in both tests (Figures 7E and 7F). It is worth noting that the activation of DRN^5HT^ and VTA^DA^ neurons in the TST is only significant after the movement onset suggesting that change in their activity was preceded by the activation of the LH→DRN and the LH→VTA pathways (Figures 7C and 7F). These results confirm that LH axon terminal activity is coincident with the postsynaptic activity of DRN^5HT^ and VTA^DA^ neurons, suggesting that the LH may be an important relay for transmitting signals to serotoninergic and dopaminergic nuclei for sensorimotor behavior control.

### Optostimulation of the LH→DRN, LH→VTA, and LH→LHb pathways is sufficient to increase mobility

**Figure 8:**
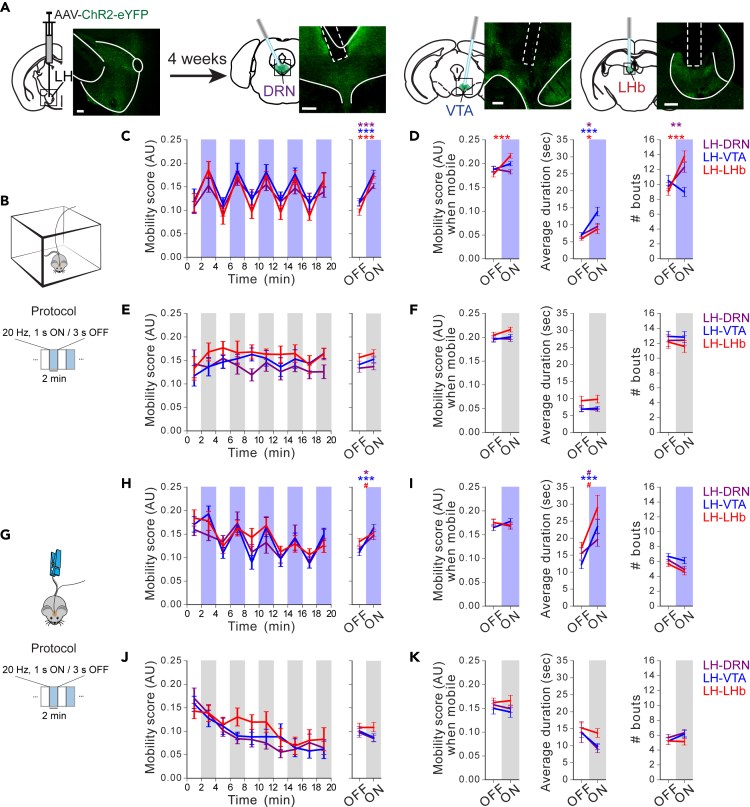
Optostimulation of the LH→DRN, LH→VTA, or LH→LHb pathway increases movement in the OFT and the TST (A) Diagram of the experimental setup and fluorescence images of ChR2-eYFP expression in LH neurons and axon terminals at the DRN, VTA, and LHb (scale bar 200 μm). (B) Diagram of the experimental protocol for the TST. The mobility of the mice was evaluated during a 20 min TST session with alternating 2-min epochs without or with 1-s, 20 Hz trains every 4 s. Mobility was automatically monitored with a video tracking system. (C) Plots of mean mobility score (mean ± SEM) during periods of optogenetic stimulation (blue) or no light (white) at the LH→DRN, LH→VTA, or LH→LHb pathways in ChR2-eYFP-expressing mice during the TST test. (D) Parameters calculated during test with optogenetic stimulation (blue) or without (white): mean mobility score during the mobility bouts (left), their duration (middle), and number (right). (E and F) Same convention as in C, D for eYFP-expressing mice. (G) Diagram of the experimental protocol for the OFT. Same pattern of the stimulation as in TST. (H–K) Same convention as in D–F for OFT test. Both tests were analyzed using four-way repeated measures ANOVA between factors of group (ChR2-eYFP- and eYFP-expressing mice), and within factors pathway (LH→DRN, LH→VTA, or LH→LHb), time period (five 4-min periods) and laser (on and off) with *post hoc* Tukey test. The p values were adjusted using the Bonferroni multiple testing correction method. 8–9 mice per group. #p < 0.1, ∗p < 0.05, ∗∗p < 0.01, ∗∗∗p < 0.001, p > 0.2 ns (not significant). See also Figures S11 and S12.

To determine whether increased activity at LH neuronal outputs is sufficient to promote motor activation, the LHs of wild-type mice were injected with AAV-ChR2-eYFP, or with an AAV encoding eYFP alone (AAV-eYFP) as a control. Optical fibers were implanted as described for the fiber photometry recordings (Figure 8A). A single optical fiber cannula was connected to a 450-nm laser light source for the optogenetic stimulation of individual LH neuronal outputs. All three LH neuronal outputs were tested using a Latin square experimental design, with 24 h between experimental sessions. The mice were subjected to 20-min OFT and TST sessions. Each session consisted of 2-min epochs with or without 5-ms light pulses delivered in 20 Hz optical stimulation trains (Figures 8B and 8G). The stimulation of individual LH neuronal outputs was sufficient to increase mobility during the 2-min stimulation epochs compared to the non-stimulation epochs in mice evaluated in the OFT and TST (Figures 8C and 8H). To examine how optical stimulation of individual LH neuronal outputs increased mobility, we evaluated the mobility score during periods when mice were mobile, indicator of movement vigor, the average duration, and the number of movement bouts with and without optical stimulation. In the OFT, we found that optical stimulation of the LH→LHb pathway increased movement vigor, but also the average duration and the number of movement bouts (Figure 8D). Conversely, optical stimulation of the LH→VTA pathway increased mobility by increasing the average duration of mobility bouts (Figure 8D), while the stimulation of the LH→DRN significantly increased the number of movement bouts (Figure 8D). In the TST, optical stimulation of all three LH neuronal outputs increased mobility by significantly increasing average duration of movement bouts, leaving unchanged movement vigor and the number of movement bouts (Figure 8I). The stimulation had no effect on the mobility of mice expressing eYFP only (Figures 8E, 8F, 8J, and 8K). These results show that increased activity at individual LH neuronal outputs is sufficient to increase mobility, largely by increasing the duration of mobile periods.

### Optostimulation of LH neuronal outputs has distinct effects in place preference and sucrose consumption

**Figure 9:**
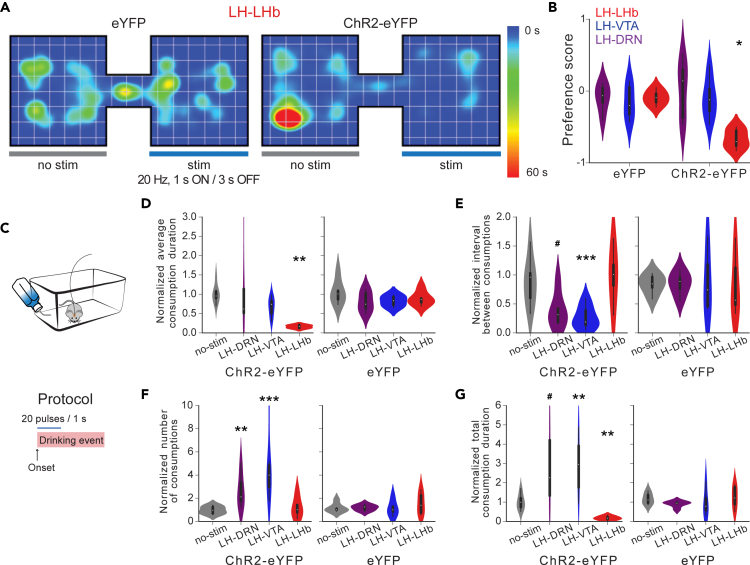
Optostimulation of the LH neuronal outputs had distinct effects in the place preference test and on the sucrose consumption (A) Representative heatmaps representing location of a mouse expressing ChR2-eYFP (left) or eYFP (right) in the RTPP. (B) The preference scores measured in the RTPP during optostimuation of the LH→DRN, LH→VTA, or LH→LHb pathways in mice expressing ChR2-eYFP or eYFP. Two-way ANOVA between factor of group (ChR2-eYFP- and eYFP-expressing mice) and within factor pathway (LH→DRN, LH→VTA, or LHA→LHb) with *post hoc* Tukey test. The p values were adjusted using the Bonferroni multiple testing correction method. (C) Diagram of the experimental protocol during sucrose consumption. A 1-s, 20 Hz optogenetic stimulation was given at the onset of a drinking event. (D–G) Plots of normalized average drinking duration (D), average interval between consumption events (E), total number of consumption events (F), and total drinking duration (G) normalized to a session without optogenetic stimulation for experimental mice expressing ChR2-eYFP (left panels) and control mice expressing eYFP only (right panels). Two-way ANOVA between factor of group (ChR2-eYFP- and eYFP-expressing mice) and within factor stimulated pathway (no stimulation, LH→DRN, LH→VTA, or LH→LHb) with *post hoc* Dunnett’s test. The p values were adjusted using the Bonferroni multiple testing correction method. 4–7 mice per stimulated pathway for the ChR2-eYFP group and 4–5 mice per stimulated pathway for the eYFP group. #p < 0.1,∗p < 0.05, ∗∗p < 0.01, ∗∗∗p < 0.001, p > 0.2 ns (not significant). See also Figures S11 and S12.

To test whether aversive or appetitive signaling at the LH neuronal outputs may contribute to control behavior responses, we have examined the effect of the optostimulation of LH neuronal outputs during a real-time place preference (RTPP) test.^35^ Consistent with previous studies, we found that the stimulation of the LH→LHb pathway was aversive. The mice avoided a context paired with the stimulation during an RTPP test (Figures 9A and 9B). However, even though the stimulation of the LH→DRN and LH→VTA pathways significantly increased mobility in the TST and OFT, it did not promote place preference or place avoidance in the RTPP. To further investigate the contribution of LH neuronal outputs in reward-related behavior responses, we examined the effect of optogenetic stimulation on sucrose consumption (Figures 9C–9G). To this end, water-deprived mice were allowed to drink the sucrose solution as described in the fiber photometry experiments, and 1-s, 20-Hz optogenetic stimulation was delivered at the onset of drinking events (Figure 9C). The effect of the optogenetic stimulation was compared to sessions without optogenetic manipulation (see STAR Methods). The optogenetic stimulation of the LH→LHb pathway at the onset of drinking events decreased average drinking duration and total consumption, which is consistent with previous findings.^31^ Conversely, optogenetic stimulation of the LH→VTA and LH→DRN pathways did not decrease drinking duration but, rather, decreased the time between drinking events, increased the total number of drinking events, and consequently increased total sucrose consumption (Figures 9D–9G). Taken together, these results suggest that LH neuronal outputs use different mechanisms to control motor activation.

## Discussion

The LH is a central nucleus that connects many brain regions that orchestrate vital behaviors.^4^^,^^5^ It is the central brain region of an interconnected network that includes the DRN, VTA, and the LHb, which all have important functions in reward and sensory processing, arousal, and motivation.^4^^,^^9^^,^^27^^,^^43^ However, most studies have focused on distinct neurochemically defined LH populations or single downstream projections,^4^^,^^5^^,^^25^^,^^26^^,^^28^^,^^44^^,^^45^ and no studies have explored how multiple LH neuronal inputs or outputs are dynamically recruited in live animals performing behavioral tasks. Here, we have examined how three major projection-defined LH neuronal outputs are dynamically engaged in tasks involving sensory integration and sensorimotor control. Our tracing experiments have shown that LH neuronal outputs to the DRN, VTA, and LHb are predominantly from distinct population projecting to a single downstream target. Multi-fiber and dual-color photometry recordings have shown that there is significant coherence in neuronal activity at LH neuronal outputs projecting to the DRN, VTA, and LHb. Increased activity at these three pathways preceded mobility onset in the TST supporting their role in promoting motor activation. While the optogenetic stimulation of individual LH neuronal outputs was sufficient to increase the mobility of mice in the OFT and TST, it had a different effect on sucrose consumption. Optogenetic stimulation of the LH→LHb pathway reduced drinking duration and total sucrose consumption, whereas optogenetic stimulation of the LH→DRN and LH→VTA pathways decreased the interval between drinking events and, consequently, increased the total number of drinking events and total sucrose consumption.

Recent studies that measured neuronal activity in multiple brain regions have shown that coordinated neuronal network dynamics are important for organizing complex behaviors.^46^^,^^47^ In line with these findings, our results show that LH neuronal outputs act in concert to transmit complementary sensory and sensorimotor signals to three major downstream brain regions to engage locomotor activation. These results further support the role of LH as a central hub to rapidly link sensory information into action, to enable exploration of an OFT, to increase spontaneous motor activity in aversive contexts such as in the TST, and likely to support escape in an active avoidance task.

Measuring activity from axon terminals in the VTA and DRN may have picked up significant signal from passing fibers, which may have resulted in significant coherence in activity from LH neuronal outputs. Our experiments using an intersectional viral approach to express calcium sensors in DRN- and VTA-projecting LH neurons support that LH axon terminals make local synaptic contact in VTA and DRN, as shown before.^21^^,^^23^^,^^24^^,^^25^^,^^26^^,^^40^ Recording activity at the cell bodies in the LH confirmed that these two projection-defined outputs show coherent activity which is unlikely to be explained solely by recording from passing fibers. Moreover, our dual-color photometry recordings have shown that activity at the axon terminals preceded changes in the activity of serotoninergic and dopaminergic neurons in the DRN and VTA, respectively, suggesting that at least some controls of postsynaptic activity are stemming from LH axons terminals. However, from our approaches, we cannot completely rule out the possibility that some signal may come from passing fibers. Moreover, while our tracing experiments have shown that a large fraction of LH neurons only project to a single downstream target, there is a small fraction of LH neurons projecting to both the DRN and VTA suggesting that coherent activity measured from axon terminals in the DRN and VTA may originate from the same LH population.

The LHb receives a net excitatory input from the LH.^28^^,^^30^ Recent work has shown that aversive stimuli are sufficient to activate the LH→LHb pathway and promote escape behavior.^29^ Moreover, this pathway encodes negative valences and rapidly develops prediction signals for negative events and aversive cues, making this pathway a critical node for value processing and avoidance learning.^28^^,^^30^ The results we obtained in our active avoidance task are consistent with these findings, providing further evidence of sensorimotor control mediated by this pathway.

The VTA receives both excitatory and inhibitory inputs from the LH, both of which play roles in motivating defensive behaviors and compulsive sucrose consumption.^23^^,^^24^^,^^25^^,^^26^^,^^40^ Glutamatergic transmission at the LH→VTA pathway (LH^Glut^→VTA) plays a role in initiating innate escape responses, signaling unexpected aversive outcomes, and signaling cues predicting aversive outcomes.^25^^,^^26^ This aversive signal is mediated by the activation of VTA dopaminergic neurons projecting to the ventromedial section of the nucleus accumbens.^25^ Barbano et al.^26^ showed that LH^Glut^→VTA transmission mediates innate escape responses by the activation of VTA glutamatergic neurons. Conversely, GABAergic transmission at the LH→VTA pathway (LH^GABA^→VTA) plays an important role in promoting behavioral activation leading to compulsive sucrose seeking and feeding,^24^^,^^40^ behaviors that are mediated by the disinhibition of VTA dopamine neurons.^24^ In light of these findings, it is likely that the increased activity observed in aversive contexts (AP, TST, active avoidance) is mediated by the LH^Glut^→VTA transmission while the increase in activity measured prior to sucrose consumption is mediated by the LH^GABA^→VTA transmission. Taken together, these results suggest that behavioral responses are promoted by an increase in the activity of DA neurons in the VTA through direct activation (aversive contexts) or disinhibition (reward-related behavior) of DA activity that originates from LH projections. We also show that the LH→DRN pathway is involved in engaging locomotor responses in freely behaving mice. Serotoninergic DRN neurons play important roles in a variety of sensorimotor, cognitive, and affective functions,^9^ involved in aversive processing,^10^ promoting active coping behaviors,^9^^,^^10^^,^^12^ or reinforcing behaviors.^13^ Here we show that the LH→DRN pathway is activated when mice are presented with a somatosensory airpuff or a mild foot shock, which is coincident with motor activation. Like the LH→VTA and LH→LHb pathways, we show that it also encodes prediction signal as well as sensory and sensorimotor signal, suggesting its contribution to organize rapid locomotion initiation.

In the DRN, serotoninergic and GABAergic neurons receive prominent inputs from the LH.^19^^,^^20^^,^^22^^,^^48^ Zhou et al.^21^ recently showed that functional excitatory and inhibitory inputs converge onto serotoninergic DRN neurons. Our *ex vivo* electrophysiological recordings showed similar inputs converging onto non-serotoninergic DRN neurons indicating that there is a complex functional relationship between the LH and the DRN.^48^ However, it remains to be determined whether this increase in serotoninergic activity is mediated by direct activation of 5-HT DRN neurons or by indirect disinhibition through local GABAergic neurons.

A recent report from Karnani et al.^8^ has demonstrated how phasic activity of hypothalamic orexin/hypocretin neurons (HONs) play a crucial role in sensorimotor control to promote rapid locomotor initiation. Using miniendoscopic *in vivo* calcium imaging, they have identified several locomotion-related HON subtypes, some of which predicted imminent locomotor initiation or engaged in sensory responses. Our results are in line with these findings. Monitoring activity from hypothalamic axon terminals in the DRN, VTA, and LHb suggests that some responses encode sensory signals, when mice were presented with airpuffs or foot shocks. We also observed increased activity during the presentation of the aversive CS in the active avoidance task, which is clearly independent of movement. On the other hand, we also measured an increase in activity preceding movement onset in the TST, suggesting that signals measured from terminals are promoting movement, which is corroborated with an increase in mobility when individual LH outputs are optogenetically stimulated. Significant correlation between neuronal activity from axon terminals and mobility onset when mice were presented with an airpuff, or during spontaneous movement in OFT, and avoidance onset in active avoidance task all suggest sensorimotor control of locomotor initiation at these three major LH neuronal outputs. Our results are also reminiscent of recent studies by the same group that directly measured the activity of HONs showing that this cell population is activated by aversive stimuli and stressors and is inhibited during food consumption, which is independent of food taste and texture.^6^^,^^34^ We propose that signal transmission from HONs in the LH plays an important role in promoting the initiation of movement by processing sensory and sensorimotor signals in the DRN, VTA, and LHb that likely contribute to organize defensive behaviors.^25^^,^^26^^,^^29^^,^^49^^,^^50^ This claim is also supported by miniendoscopic *in vivo* calcium imaging of LHb neurons in mice responding in looming experiments which revealed that specific LHb neuron ensembles are active before the mice start running away from an aversive threat,^45^ suggesting that some of the excitatory inputs driving these neuronal ensembles may be provided by HONs.^8^ Since most orexin-expressing neurons in the LH co-release glutamate,^51^^,^^52^ it would be interesting to determine how excitatory and orexigenic transmissions drive activity in the DRN, VTA, and LHb and, as a result, control behaviors.

Although the optogenetic stimulation of individual LH neuronal outputs increased mobility, optogenetic stimulation at drinking onset in the SCT had a different effect. Indeed, the activation of the LH→LHb pathway at drinking onset decreased drinking duration and total sucrose consumption, whereas the activation of the LH→DRN or the LH→VTA pathway increased total sucrose consumption by decreasing the time interval between drinking periods. One possible underlying mechanism that enhances sucrose consumption following LH→VTA activation may be mediated by the co-release of neurotensin from LH axon terminals in the VTA, a neuropeptide that promotes reward seeking by enhancing glutamate transmission in the VTA.^53^ In the SCT, phasic activity at the LH^GABA^→VTA pathway at the onset of sucrose consumption may also be sufficient to motivate reward-seeking behaviors by decreasing time of the interval between drinking periods.^40^^,^^44^

In summary, our findings show how coherent signals are transmitted to the LH→DRN, LH→VTA, and the LH→LHb pathways for sensorimotor control. For many contexts and stimuli, the responses of the LH→DRN, LH→VTA, and LH→LHb pathways are coherent, suggesting that the LH conveys complementary sensory and sensorimotor signals that engage several downstream brain regions to promote locomotor activation. It is likely that distinct 5-HT DRN, and DA VTA neuronal populations, segregated by their inputs and outputs, play different but complementary roles in cue processing and adaptive behaviors. Another interesting question that remains to be investigated is whether the three distinct LH neuronal populations, independently projecting to DRN, VTA, and LHb, receive common or distinct inputs from upstream brain regions.

### Limitations of the study

In this study, we have expressed the calcium sensor GCaMP6s in the LH of wild-type mice to monitor calcium dynamics simultaneously at LH axon terminals in the DRN, VTA, and LHb. This approach does not allow us to make distinction whether the signals originating from the LH in the target areas are from any specific cell population in the LH, beside their projection specificity. We suspect that the signal we monitored may largely come from HON neurons as detailed in the discussion, but potential contribution of other LH neuronal type in the transmission of sensorimotor signal may also be important. Another limitation of this study, inherent of the fiber photometry imaging, in which bulk calcium dynamics is measured, does not allow to discern if there exist two or more LH subpopulations which may have different functional dynamics. Viral intersectional strategy to express calcium sensor in projection-defined LH subpopulation for miniscope calcium imaging would be required to examine if there are functionally distinct LH populations signaling sensorimotor information to the DRN, VTA, and the LHb.

## STAR★Methods

### Key resources table

**Table:** 

REAGENT or RESOURCE	SOURCE	IDENTIFIER
**Antibodies**		

Sheep polyclonal anti-tryptophane hydroxylase	Millipore	Cat# AB1541; RRID:AB_90754
Sheep polyclonal anti-tyrosine hydroxylase	Millipore	Cat# AB1542; RRID:AB_90755
Donkey Anti-Sheep IgG DyLight 405-AffiniPure	Jackson ImmunoResearch Labs	Cat# 713-475-147; RRID:AB_2340740

**Bacterial and virus strains**		

AAVDJ-CAG-GCaMP6s	CERVO Canadian Optogenetics and Vectorology Foundry Core Facility	RRID:SCR_016477
AAV9-hSyn-eYFP	CERVO Canadian Optogenetics and Vectorology Foundry Core Facility	RRID:SCR_016477
AAV9-CAG-DIO-jrGECO1a	CERVO Canadian Optogenetics and Vectorology Foundry Core Facility	RRID:SCR_016477
AAV9-EF1a-fDIO-GCaMP6s	CERVO Canadian Optogenetics and Vectorology Foundry Core Facility	RRID:SCR_016477
AAV9-hSyn-ChR2(H134R)-eYFP	CERVO Canadian Optogenetics and Vectorology Foundry Core Facility	RRID:SCR_016477
retroAAV-CAG-flpO-P2A-TagBFP2	CERVO Canadian Optogenetics and Vectorology Foundry Core Facility	RRID:SCR_016477
retroAAV-CAG-cre-P2A-mNeptune	CERVO Canadian Optogenetics and Vectorology Foundry Core Facility	RRID:SCR_016477

**Chemicals, peptides, and recombinant proteins**		

Cholera Toxin Subunit B (Recombinant), Alexa Fluor™ 488 Conjugate	Invitrogen: ThermoFisher Scientific	Cat: C34776
Cholera Toxin Subunit B (Recombinant), Alexa Fluor™ 647 Conjugate	Invitrogen: ThermoFisher Scientific	Cat: C34778
Cholera Toxin Subunit B (Recombinant), Alexa Fluor™ 555 Conjugate	Invitrogen: ThermoFisher Scientific	Cat: C34775
Tetrodotoxin citrate, Na+ channel blocker (TTX)	Abcam Canada	Cat: ab120055
4-Aminopyridine (4-AP)	ThermoFisher Scientific	Cat: AC104570050; CAS: 504-24-5
Biocytin (ε-Biotinoyl-L-Lysine)	ThermoFisher Scientific	Cat: B1592
NBQX disodium salt	Alomone labs	Cat: N-186; Cat:479347-86-9
Gabazine hydrobromide	Alomone labs	Cat: G-216; CAS: 104104-50-9

**Deposited data**		

Github repository for all the codes and data related to the manuscript	This paper	https://github.com/katemartian/LHoutputsManuscript
Master source data	This paper	https://github.com/katemartian/LHoutputsManuscript/tree/master/sourceData
Python package used to process, analyze, and visualize fiber photometry data	This paper	https://github.com/katemartian/LHoutputsManuscript/blob/master/FiberPhotometryDataAnalysis.ipynb
Optogenetic data	This paper	https://github.com/katemartian/LHoutputsManuscript/blob/master/rawData/LHoutputs_opto_dlc.ipynb
Master raw data	This paper	https://github.com/katemartian/LHoutputsManuscript/tree/master/rawData

**Experimental models: Organisms/strains**		

Mouse : ePet-cre (B6.Cg-Tg(Fev-cre)1Esd/J)	Jackson Laboratories	Strain #:012712; RRID:IMSR_JAX:012712
Mouse : DAT-ires-cre (B6.SJL-*Slc6a3*^*tm1.1(cre)Bkmn*^/J)	Jackson Laboratories	Strain #:006660; RRID:IMSR_JAX:006660
Mouse : C57BL/6NCrl	Charles River Laboratories	Strain Code 027

**Software and algorithms**		

Med-PC	Med Associates	RRID:SCR_012156
ANY-maze version 6.1	Stoelting Co.	RRID:SCR_014289
DeepCutLab	https://github.com/DeepLabCut/DeepLabCut	RRID:SCR_021391
Bonsai	https://bonsai-rx.org/	RRID:SCR_017218
ImageJ	https://imagej.net/	RRID:SCR_003070
Python 3.7	Python Software Foundation	https://www.python.org/
Google CoLab	https://colab.research.google.com/	RRID:SCR_018009
Affinity designer	Serif	RRID:SCR_016952
pClamp 11.2.1.00	Molecular Devices	RRID:SCR_011323

**Other**		

Optical fiber cannula	Home made	NA
Branching patchcord for fiber photometry recordings	Doric Lenses	BFP(3)_200/220/900-0.37_2m_FCM-3xMF1.25
Mono fiberoptic patchcord	Doric Lenses	MFP_200/220/900-0.37_2m_FC-MF1.25
Real-time place preference (RTPP) test box	Custom : Plastique Multifab	NA
450-nm laser Diode	Doric Lenses	LDFLS_450/075
Open field (OFT) arena	Custom : Plastique Multifab	NA
Tail suspension test apparatus	Custom : Plastique Multifab	NA
3-Channel Multi-fiber photometry system	Neurophotometrics Ltd	FP3001
Custom built Multi-fiber photometry system	Kim, C. K. et al.^5^	NA
Shuttle box	Med Associates	CR-ENV-010MC-X3

### Resource availability

#### Lead contact

Further information and requests for resources should be directed to and will be fulfilled by the lead contact Christophe Proulx (Christophe.proulx@fmed.ulaval.ca).

#### Material availability

This study did not generate new unique reagents.

#### Data and code availability

All the code and dataset are publicly accessible. We have created a Github repository (https://github.com/katemartian/LHoutputsManuscript) to store all the codes and data related to the manuscript. In the repository, one can find the raw data and results of the analysis from all our fiber photometry recordings and optogenetic manipulations in hierarchical data format, h5 files (https://github.com/katemartian/LHoutputsManuscript/tree/master/rawData), python package used to process, analyze, and visualize fiber photometry data (https://github.com/katemartian/LHoutputsManuscript/blob/master/FiberPhotometryDataAnalysis.ipynb), jupyter notebook used to analyze optogenetics data (https://github.com/katemartian/LHoutputsManuscript/blob/master/rawData/LHoutputs_opto_dlc.ipynb), source data (https://github.com/katemartian/LHoutputsManuscript/tree/master/sourceData), and jupyter notebooks used to analyze the data from raw files acquired using fiber photometry systems, ANY-maze, Med Associates, and DeepLabCut to final plots presented in the figures and final excel source data files used for statistical analysis (https://github.com/katemartian/LHoutputsManuscript/tree/master/rawData). One can download the data (h5 files) and codes (jupyter notebooks), and replicate the whole analysis on their machine using python, or copy the files into their Google Drive and use Google Colab (https://colab.research.google.com/) to run the codes. Alternatively, one can see the outputs of code cells of the jupyter notebooks without executing code.

Any additional information required to reanalyze the data reported in this work is available from the lead contact upon request.

### Experimental model and study participant details

Both males and females were used in this study (20-30 g). All experiments were performed with 8- to 12-week-old wild-type C57Bl/6, ePet-cre, or DAT-ires-cre mice. The mice were housed 2-4 per cage and were kept on a 12 h/12 h light/dark cycle. All the experiments were performed in accordance with the Canadian Guide for the Care and Use of Laboratory Animals guidelines and were approved by the Université Laval Animal Protection Committee.

### Method details

#### Stereotactic injections

The mice were anesthetized with isoflurane for the stereotaxic injections of adeno-associated viruses (AAVs) and the implantation of optical fibers. Viral titers ranged from 10^12^ to 10^13^ genome copies per milliliter and volumes ranged from 100 to 200 nl per side. Injections were performed using a glass pipette mounted on a stereotactic table. The AAVs were infused at a rate of 1 nl/sec. At the end of the injection, the pipet was left *in situ* for 5 min to allow the virus to diffuse into the surrounding tissue. Three to four weeks later, for the fiber photometry and optogenetic experiments, the mice were implanted with 200- or 400 μm cannulae with a metal ferrule for the fiber photometry recordings, and 200 μm cannulae with a ceramic ferrule for the optogenetic manipulations.

For most fiber photometry experiments, the mice were bilaterally injected with AAVDJ-CAG-GCaMP6s in the LH, and optical fiber cannulae were implanted with their tips immediately above the DRN, VTA, and LHb. For the intersectional viral strategy, the mice were injected with retroAAV-CAG-cre-P2A-mNeptune in the VTA, retroAAV-CAG-flpO-P2A-TagBFP2 in the DRN, and a mix of the AAV9-CAG-DIO-jrGECO1a and AAV9-EF1a-fDIO-GCaMP6s in the LH, and an optical fiber was chronically implanted above the LH. For the dual-color fiber photometry, ePet-cre mice were injected with AAVDJ-CAG-GCaMP6s in the LH and AAV9-CAG-DIO-jrGECO1a in the DRN, and optical fiber was implanted above the DRN. Similarly, DAT-ires-cre mice were injected with AAVDJ-CAG-GCaMP6s in the LH and AAV9-CAG-DIO-jrGECO1a in the VTA, and optical fiber was implanted above the VTA.

For the optogenetic experiments, the mice were bilaterally injected with AAV9-hSyn-hChR2(H134R)-eYFP or AAV9-hSyn-eYFP in the LH, and optical fibers were implanted above the DRN, VTA, and LHb. The mice were tested 2 weeks post-cannulae implantation to give them time to recover from the surgical procedures.

For the electrophysiology experiments, the mice were injected with AAV9-hSyn-hChR2(H134R)-eYFP in the LH. Acute brain slices were prepared 3 weeks later for whole-cell patch clamp recordings.

To label DRN-, VTA-, and LHb-projecting neurons in the LH of the same animal, the mice were injected with 100 nl of CTb488 (Alexa Fluor 488-conjugated cholera toxin subunit B), CTb594, and CTb647 in the DRN, VTA, and LHb, respectively. The mice were transcardially perfused 3 days later, and their brains were processed for histology.

The coordinates for the injections were as follows: LH : -1.2 mm AP, ±1.0 mm ML, -5.2 mm DV; LHb : -1.65 mm AP, ±0.45 mm ML, -2.8 mm DV; VTA : -3.3 mm AP, ±0.5 mm ML, -4.8 mm DV; DRN : -4.65 mm AP, 0.0 mm ML, -3.2 mm DV. The coordinates for implantation were the following: LH : -1.2 mm AP, -1.0 mm ML, -5.0 mm DV; LHb: -1.65mm AP, -0.45mm ML, -2.4mm DV; VTA: -3.3 mm AP, 1.0 mm ML, -4.4 mm DV with 10° angle; DRN: -4.65 mm AP,-1.05 mm ML, -2.9 mm DV with 20° angle.

#### Fiber photometry recordings

A custom-build^54^ or a Neurophotometrics fiber photometry system was used to record calcium signals. Both systems had the same set of dichroic mirrors, filters, and LEDs. Light from 415-nm, 470-nm, and 560-nm LEDs were bandpass filtered, collimated, reflected by dichroic mirrors, and focused by a 20× objective (numerical aperture, NA 0.39). The light passed through a patch cord of three fibers, that were connected to the implanted cannulae. The emitted fluorescence was collected by the same fibers, filtered, and separated into red and green images, which were projected on a CMOS camera sensor. The excitation power was adjusted so as to get 50 to 70 uW of each of the lights at the tip of the patch cord. The custom-build system was controlled using LabJack and a custom-written Matlab code. The Neurophotometrics system was run by Bonsai open-source software. For most of the experiments, light from 415-nm- and 470-nm LEDs was alternated such that the camera captured fluorescent excitation light from either the 415-nm or 470-nm LED. The camera captured images at 20 Hz. Signals from the two excitation wavelengths were sampled at 10 Hz. For the experiments with the ePet-cre and DAT-ires-cre mice, the 560-nm LED was alternated with the 470-nm LED. For the intersectional viral strategy experiments, all three (415-nm, 470-nm, and 560-nm) LEDs were alternated such that the camera captured one light at a time. See^33^ for more details. The mice were connected to a 3-fiber patch cord to record the signals during all the tests. For intersectional viral strategy and experiments with the ePet-cre and DAT-ires-cre mice, only one fiber was connected.

##### Airpuff test

The mice were placed in the open field arena and airpuffs were delivered on top of the animals every 50 to 70 s, for a total of 5 airpuffs per 6 min session. Each airpuff was delivered on top of the animal head from about 30 cm. Airpuff delivery was paired with a key press on a computer keyboard that was registered by the fiber photometry software and that provided timestamps of the airpuffs. Mice were tested several times and total of 5-20 events were recorded with each mouse.

##### Sucrose consumption test

The mice were water-deprived for 24 h before the experiment. For the test, the mice were placed in a cage with free access to a spout delivering a small amount of sucrose solution. Consumption periods were automatically tracked with a custom lickometer. The setup to measure licks consisted of a mouse cage covered with a metal grid floor. The cage was equipped with a copper wire-wrapped metal sipper tube from which sucrose solution was delivered. Each lick closed an electrical circuit for the duration of the contact with the sipper tube. The junction potential between the metal sipper tube and the mouse was recorded using the ANY-maze system. ANY-maze provided timestamps of each of the loop closures. To align the lick timestamps with the fiber photometry recordings, the start of the ANY-maze test was either triggered by the fiber photometry system, or ANY-maze and fiber photometry software were used on the same computer and recorded real timestamps.

##### Tail suspension test

The mice were suspended by their tail for 10 min. Middle point of the mouse was tracked using a camera and ANY-maze video-tracking software and speed was calculated by ANY-maze. The ANY-maze recordings were aligned to the fiber photometry recordings using the same strategy as with the sucrose consumption test.

##### Open field test

The mice were placed in an open field (50 cm x 50 cm) and speed was tracked same way as in the tail-suspension test.

##### Avoidance test

A mouse was placed in a two-compartment shuttle box chamber (Med Associates). A conditioned stimulus (CS, light, and tone) was provided for 8 s pseudo-randomly with an average ITI of 40 s. At the end of the 8-s CS, a mild foot-shock (0.2 mA) was delivered through the grid floor for 8 s or until the mouse crossed to the other compartment, which stopped the shock. If the mouse crossed within the 8-s CS, no shock was delivered. This was referred as avoidance. The mice were tracked using a camera and ANY-maze video-tracking software. To align the recordings of all the setups, Med-PC V (Med Associates) software triggered the start of the fiber photometry and the ANY-maze software at the beginning of the tests. Recordings were done on the first (early) and the fifth (late) day of the training. Due to the light change in the chamber during the experiments, ANY-maze failed to track the mice. Therefore, the position of the mice was analyzed post hoc using DeepLabCut, a toolbox for markerless animal pose estimation.^38^

#### Optogenetic manipulations

For the optogenetic experiments, the mice were connected to a 450-nm laser (Doric Lenses) through an optical fiber and a rotary joint. Pulses of blue light were controlled by the ANY-maze software. The stimulation protocol was 20-Hz trains and 5-ms pulses for 1 s every 4 s. The light intensity was adjusted to provide 10 mW at the tips of the implanted optical fiber cannulae. During a trial, the mice were connected to a single optical fiber cannula implanted above the DRN, VTA, or LHb. For each test, the mice were tested on consecutive days using a Latin square experimental design. The same group of mice were used in the real-time place preference, the tail suspension test, the open field test, and the sucrose consumption test.

##### Real-time place preference test

The mice were placed in a chamber with two compartments connected by a small corridor. After 1 min of habituation, one of the compartments was paired with an optogenetic stimulation. A mouse received a photostimulation every time it entered the paired chamber (randomly assigned). To maximize novelty and exploratory behavior on consecutive testing days, the RTPP apparatus was used as follows: day 1 with a plain floor, day 2 with bedding covering the floor, and day three with a powder of finely grounded food pellet on the floor. No significant bias has been observed in any condition (plain floor, bedding, finely ground food pellet; please see the source data Fig8 stats.ipynb) showing that animal’s chamber preference was not alternated due to food presence). Stimulation chambers were randomly assigned on each of the three days of testing. The location of the mouse (chamber 1, chamber 2, or corridor) was tracked, and laser activation was controlled using the ANY-maze video-tracking system.

##### Tail suspension test and open field test

The mice were suspended by their tail or were placed in an open field for 20 min. The photostimulation was alternated between a 2-min periods without stimulation and with a 2-min period with photostimulation trains (20 Hz, 1 second, 5-ms pulse duration, every 4 seconds). The position of the mice was analyzed post hoc using DeepLabCut.

##### Sucrose consumption test

The same protocol used for the fiber photometry recordings was used for optogenetic manipulations. The ANY-maze tracking system detected drinking onset and triggered 1s 20Hz laser photostimulation for each drinking event. To define the baseline of drinking behavior for each animal, the mice were tested 3 times (without photostimulation) prior to the photostimulation sessions (pre-sessions), and 1 time after (without photostimulation, post-session). The DRN, VTA, and LHb photostimulation sessions and a session with no photostimulation were alternated using a Latin square experimental design.

#### Electrophysiology

Three weeks after the injection of AAV-ChR2-eYFP in the LH, the mice were anesthetized with isoflurane and were perfused transcardially with 10 mL of ice-cold NMDG-artificial cerebrospinal fluid (aCSF) solution containing (in mM): 1.25 NaH2PO4, 2.5 KCl, 10 MgCl2, 20 HEPES, 0.5 CaCl2, 24 NaHCO3, 8 D-glucose, 5 L-ascorbate, 3 Na-pyruvate, 2 thiourea, and 93 NMDG (osmolarity was adjusted to 300–310 mOsmol/L with sucrose). The pH was adjusted to 7.4 using 10 N HCl. Kynurenic acid (2 mM) was added to the perfusion solution on the day of the experiment. The brains were then quickly removed, and 250 μm acute brain slices encompassing the DRN were prepared using a Leica VT1200S vibratome. The slices were placed in a 32°C oxygenated perfusion solution for 10 min and were then incubated for 1 h at room temperature in HEPES-aCSF solution (in mM): 1.25 NaH2PO4, 2.5 KCl, 10 MgCl2, 20 HEPES, 0.5 CaCl2, 24 NaHCO3, 2.5 D-glucose, 5 L-ascorbate, 1 Na-pyruvate, 2 thiourea, 92 NaCl, and 20 sucrose (osmolarity was adjusted to 300–310 mOsmol/L with sucrose). The pH was adjusted to 7.4 using 10 N HCl. They were then transferred to a recording chamber on the stage of an upright microscope (Zeiss) where they were perfused with 3-4 mL/min of aCSF (in mM): 120 NaCl, 5 HEPES, 2.5 KCl, 1.2 NaH2P04, 2 MgCl2, 2 CaCl2, 2.5 glucose, 24 NaHCO3, and 7.5 sucrose). The perfusion chamber and the aCSF were kept at 32°C. All the solutions were oxygenated with 95% O2/5% CO2. A 60× water immersion objective and a video camera (Zeiss) were used to visualize neurons in the DRN. Borosilicate glass (3-7 MΩ resistance) recording pipettes were pulled using a P-1000 Flaming/ Brown micropipette puller (Sutter Instruments). Recordings were performed using an Axopatch 200B amplifier (Molecular Devices). For the voltage-clamp recordings, the intracellular solution consisted of (in mM): 115 cesium methanesulfonate, 20 cesium chloride, 10 HEPES, 2.5 MgCl2, 4 Na2ATP, 0.4 Na3GTP, 10 Na-phosphocreatine, 0.6 EGTA, and 5 QX314, as well as 0.2% biocytin (pH 7.35). For the current-clamp recordings, the intracellular solution consisted of (in mM): 130 K-gluconate, 5 KCl, 10 HEPES, 2.5 MgCl2, 4 Na2ATP, 0.4 Na3GTP, 10 Na-phosphocreatine, and 0.6 EGTA (pH 7.35). Signals were filtered at 5 kHz using a Digidata 1500A data acquisition interface (Molecular Devices, San Jose, CA) and acquired using pClamp 10.6 software (Molecular Devices). Pipette and cell capacitance were fully compensated. To examine monosynaptic transmission, the extracellular recording solution was supplemented with 1 μM TTX and 100 μM 4-AP. For the voltage-clamp experiments, postsynaptic currents were measured in DRN neurons clamped at -60 mV and 0 mV holding voltage following optogenetic stimulation of LH axon terminals with 5-ms blue light pulses delivered through the objective with a Colibri 7 LED light source (Zeiss). Excitatory and inhibitory transmissions were blocked with 3 mM NBQX and 10 mM gabazine, which are AMPA and GABA-a receptor antagonists, respectively. For the current-clamp experiments, DRN neurons were depolarized at -30mV and changes in the postsynaptic potential were measured before and after the addition of 10 mM gabazine. Once the recordings were completed, the slices were fixed in 4% formaldehyde for 30 min and were then transferred to a 0.1M phosphate buffer solution for post hoc histological analysis.

#### Histology and immunostaining

The mice were deeply anesthetized using a mix of ketamine/xylazine (100 and 10 mg/kg, respectively, intraperitoneally) and were transcardially perfused with saline followed by a 0.1 M phosphate buffer solution (PB, pH7.4) containing 4% paraformaldehyde. The brains were postfixed overnight in the same solution, rinsed with PB, and stored in PB. Brain sections (100 μm for histology and 50 μm for immunostaining) were cut with a vibratome along the coronal plane.

Sections used for fiber photometry recordings and optogenetic manipulations were examined to confirm injection sites and cannulae placements. Recordings were excluded post hoc in the rare cases where an optical fiber was misplaced or where the expression of the construct of interest was off-target or low.

DRN sections from ePet-cre mice and VTA sections from DAT-ires-cre mice were stained for TPH (tryptophan hydroxylase, a marker for 5-HT neurons) and for TH (tyrosine hydroxylase, a marker for DA neurons), respectively, using a standard 2-day immunostaining protocol. Briefly, free-floating slices were first blocked in PB containing 10% normal donkey serum (NDS) and 0.2% Triton X-100 for 1 h. They were then incubated overnight with primary antibodies diluted in PB containing 3% NDS and 0.2% Triton X-100 and then with a secondary antibody diluted in PB containing 3% NDS for 2 h at RT. The primary antibodies were anti-TPH (Millipore, sheep polyclonal, 1:1000 dilution) and anti-TH (Millipore, sheep polyclonal, 1:1000 dilution). The secondary antibody was donkey anti-sheep IgG DyLight 405 (Jackson ImmunoResearch, 1:500 dilution). Sections from the electrophysiological recordings were immunostained for TPH using the protocol described above. Immunostained sections and sections from mice injected with CTb were mounted using FluoromountTM Aqueous Mounting Medium (Millipore-Sigma) and imaged using a Zeiss LSM700 confocal microscope. CTb-positive cells were counted using the ImageJ plugin Colocalization Object Counter and semi manually registered to the Allen Brain Atlas using the ImageJ plugin BigWarp. Cells were counted in the LH of hemisphere ipsilateral to the injection sites.

#### Data analysis

##### Fiber photometry data

To store, process, analyze, and visualize our fiber photometry data, a python package was created (https://github.com/katemartian/LHoutputsManuscript/blob/master/FiberPhotometryDataAnalysis.ipynb). We have created jupyter notebooks containing the analysis pipeline for each group of mice: expressing GCaMP6s for the APT and SCT (https://github.com/katemartian/LHoutputsManuscript/blob/master/rawData/LHoutputs_apt-sct.ipynb), expression GCaMP6s for the OFT and TST (https://github.com/katemartian/LHoutputsManuscript/blob/master/rawData/LHoutputs_oft-tst.ipynb), expressing eYFP (https://github.com/katemartian/LHoutputsManuscript/blob/master/rawData/LHoutputs_eYFP.ipynb), intersectional approach (https://github.com/katemartian/LHoutputsManuscript/blob/master/rawData/LHoutputs_retro.ipynb), avoidance task (https://github.com/katemartian/LHoutputsManuscript/blob/master/rawData/LHoutputs_avoidanceTask.ipynb), ePet-cre (https://github.com/katemartian/LHoutputsManuscript/blob/master/rawData/LHoutputs_5HT.ipynb) and DAT-cre mice (https://github.com/katemartian/LHoutputsManuscript/blob/master/rawData/LHoutputs_DA.ipynb).

To calculate the standardized *dF/F* (*z-score dF/F*, *zdF/F*), the algorithm described in^33^ was used. Briefly, calcium-dependent (*signals*) and calcium-independent (*references*) traces were smoothed using a band-pass filter (moving average was used in^33^), flattened by removing the baseline using an airPLS algorithm (adaptive iteratively reweighted Penalized Least Squares^55^), and standardized to a mean value of 0 and a standard deviation of 1. *References* were fitted to *signals* using a non-negative robust least squares regression (Lasso algorithm), and *zdF/F* was calculated by subtraction from standardized *signals* and fitted *references*.

To define the onsets and offsets of consumption, lickings recorded with the lickometer were used: onsets were defined as licks that persisted for at least 0.5 s, and offsets defined as the absence of licks for at least 1 s.

For most of the tests, ANY-maze provided the speed of the mice over time. For the avoidance conditioning test, the coordinates of the mice over time were exported from videos using the DeepLabCut toolbox,^38^ and the speed was calculated using the following formula: (speed = √((x1-x0)^2^ + (y1-y0)^2^)/(t1-t0). To account for different tracking setups and experimental setups, all the speeds were transformed to a standardized range of [0, 1] (mobility score). Mobility score was used to define mobility and immobility onsets. An immobility bout was defined as a period where the values were < 0.1 and lasted at least 2 s. Mobility bouts were defined as the periods outside of immobility bouts.

*zdF/F* was aligned and averaged around the specific events such as airpuff, consumption onset, mobility onset, CS, for each animal across all trials. Not all animals, trials, pathways were included at this step of the analysis, only the ones with high signal to noise ratio, which were defined by experimenter. Jupyter notebooks (https://github.com/katemartian/LHoutputsManuscript/blob/master/rawData) contain the information about the recordings that were taken for further analysis after *zdF/F* calculation.

To measure the change from a baseline, area under the curve (AUC) was calculated. For airpuff, baseline AUC was calculated at the time frames -2 – -1 s, airpuff at 0 – 1.5 s. For consumption onset, baseline AUC was calculated at the time frames -2 – -1 s, onset at -0.5 – 1 s, drinking – at 2 – 4 s. For mobility onset, immobile AUC was calculated at the time frames -3 – -1 s, pre-onset at -1 – 0 s, onset at 0 – 1 s, and mobility at 1 – 3 s. In avoidance test, baseline AUC was calculated at the time frames -2 – -1 s, CS at 0 – 8 s, crossed at 8–11 s. AUC was normalized to the duration of the time frames (*AUCnorm* = *AUC*/(*t1 − t0*), where t0 and t1 are the beginning and the end of the time frames respectively).

Similarly, mobility score was aligned and averaged around the events, and AUC was calculated. For correlation analysis between calcium signal and mobility score, traces were interpolated to the same frequency.

##### Optogenetics data

All optogenetic data was exported from ANY-maze and analyzed using custom python codes (https://github.com/katemartian/LHoutputsManuscript/blob/master/rawData/LHoutputs_opto_dlc.ipynb). For the RTPP, ANY-maze data of laser activity over time was exported from ANY-maze and the preference score was calculated using the following formula: Preference score = (time during laser ON – time during laser OFF) / total time as detailed before.^35^

For the OFT and TST, the speeds tracked by ANY-maze were transformed into a standardized range [0, 1] (mobility score) to maintain the same format as the mobility score measured along with the fiber photometry recordings. Average mobility scores were calculated for periods of laser ON and OFF. Mobility bouts were defined as described above, their duration, number, and mobility score during these bouts were calculated for periods of laser ON and OFF.

For the SCT, ANY-maze data of licks recorded over time were exported, and the consumption onsets and offsets were found as described above for fiber photometry recordings. The following parameters were calculated: average consumption duration, average interval between events, number of events, and total consumption duration for three pre-sessions without photostimulation, sessions with photostimulation, and one post-session without stimulation. As the last pre-session and the post-session had approximately the same values for the parameters measured, these values were averaged and were used for the data normalization of values acquired during sessions with photostimulation.

### Quantification and statistical analysis

Statistical analyses were performed using R programming language. The codes were saved in jupyter notebooks and available along with the rest of the source data and can be found in our GitHub repository (https://github.com/katemartian/LHoutputsManuscript/tree/master/sourceData). To open, jupyter notebooks do not require any software installation, they can be opened directly in the GitHub repository (https://github.com/katemartian/LHoutputsManuscript) (e.g. statistical analysis for Figure 1 (https://github.com/katemartian/LHoutputsManuscript/blob/master/sourceData/Fig1_stats.ipynb)) or using Google Colab (https://colab.research.google.com/) (e.g. statistical analysis for Figure 1 (https://colab.research.google.com/github/katemartian/LHoutputsManuscript/blob/master/sourceData/Fig1_stats.ipynb)). Each notebook contains code cells, showing the pipeline of our analysis and cell outputs, showing the results of statistical tests. So one can see all the details of statistical analysis without executing the code. The summary of the statistical analysis is also available in each excel source data files.

Data distributions were tested using the Shapiro-Wilk normality test. Parametric or nonparametric tests were chosen depending on the number of observations, the distribution, and the model. For most of the data, a multi-factor mixed ANOVA with post hoc Dunnett’s or Tukey tests was used. We used a two-tailed Chi-square test to assess the observed frequencies of occurrence for each LH populations projecting to one or multiple outputs assuming that all populations had the same probability of occurrence (1/7), with Bonferroni adjustment of the p values. The exact tests are indicated in the figure legends. Significance was set at p < 0.05 in most tests. Electrophysiological and behavior experiments were replicated at least three times.

#### Sample size

The sample size for each group for behavioral, anatomical, and electrophysiological experiments was determined from previously published work and from pilot experiments performed in our laboratory.
