# Supplementary material for: Hypothalamic neuronal outputs transmit sensorimotor signals at the onset of locomotor initiation
Source: iScience. 2023 Oct 24;26(11):108328. doi: 10.1016/j.isci.2023.108328 (PMC10665817; doi:10.1016/j.isci.2023.108328)
Supplement: Document S1. Figures S1–S12 [file mmc1.pdf]

iScience, Volume *26*

Supplemental information

Hypothalamic neuronal outputs transmit sensorimotor signals at the onset of locomotor initiation

Ekaterina Martianova, Renata Sadretdinova, Alicia Pageau, Nikola Pausic, Tommy Doucet Gentiletti, Danahé Leblanc, Arturo Marroquin Rivera, Benoît Labonté, and Christophe D. Proulx

Supplemental figure legends

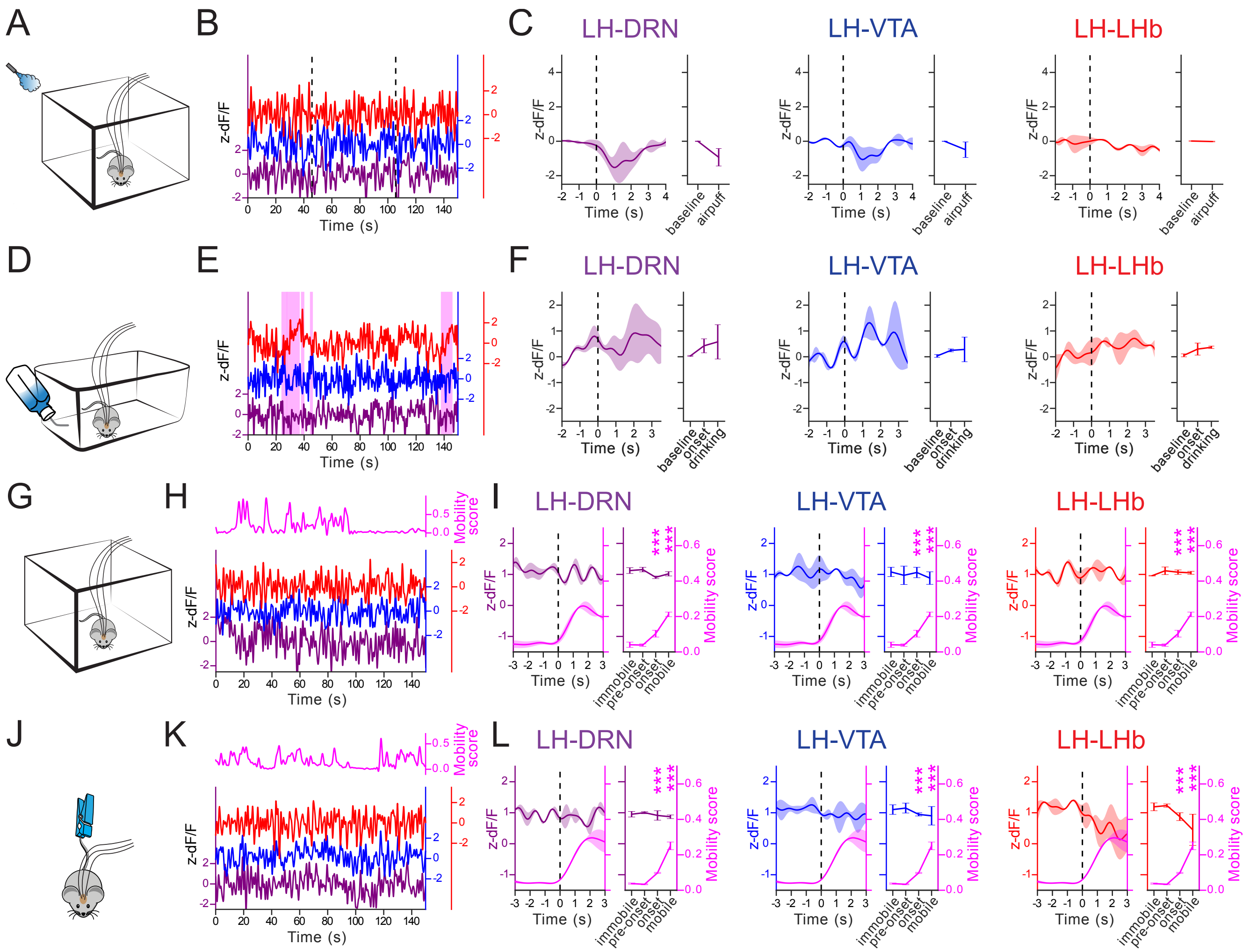

Figure S1. Recordings from the LH→DRN, LH→VTA, and LH→LHb pathways of control eYFP-expressing mice. Related to Figure 2. (**A**) Diagram of the experimental setup for the airpuff. (**B**) Representative signal traces associated to the airpuffs (dashed vertical bars) simultaneously measured at the LH→DRN, LH→VTA, and LH→LHb pathways. (**C**) Peri-event plot of the average signals to all the airpuff events at the LH→DRN, LH→VTA, and LH→LHb pathways. Plot for average response before and after airpuffs. Lines represent mean ± SEM. Same convention as **A-C** for sucrose consumption test (**D-F**), open field test (**G-I**), and tail suspension test (**J-L**). The sucrose consumption events are represented by pink shaded box in **E**. The magenta lines are the mobility scores. The statistical analysis was performed along with the data from the mice expressing GCaMP6s.

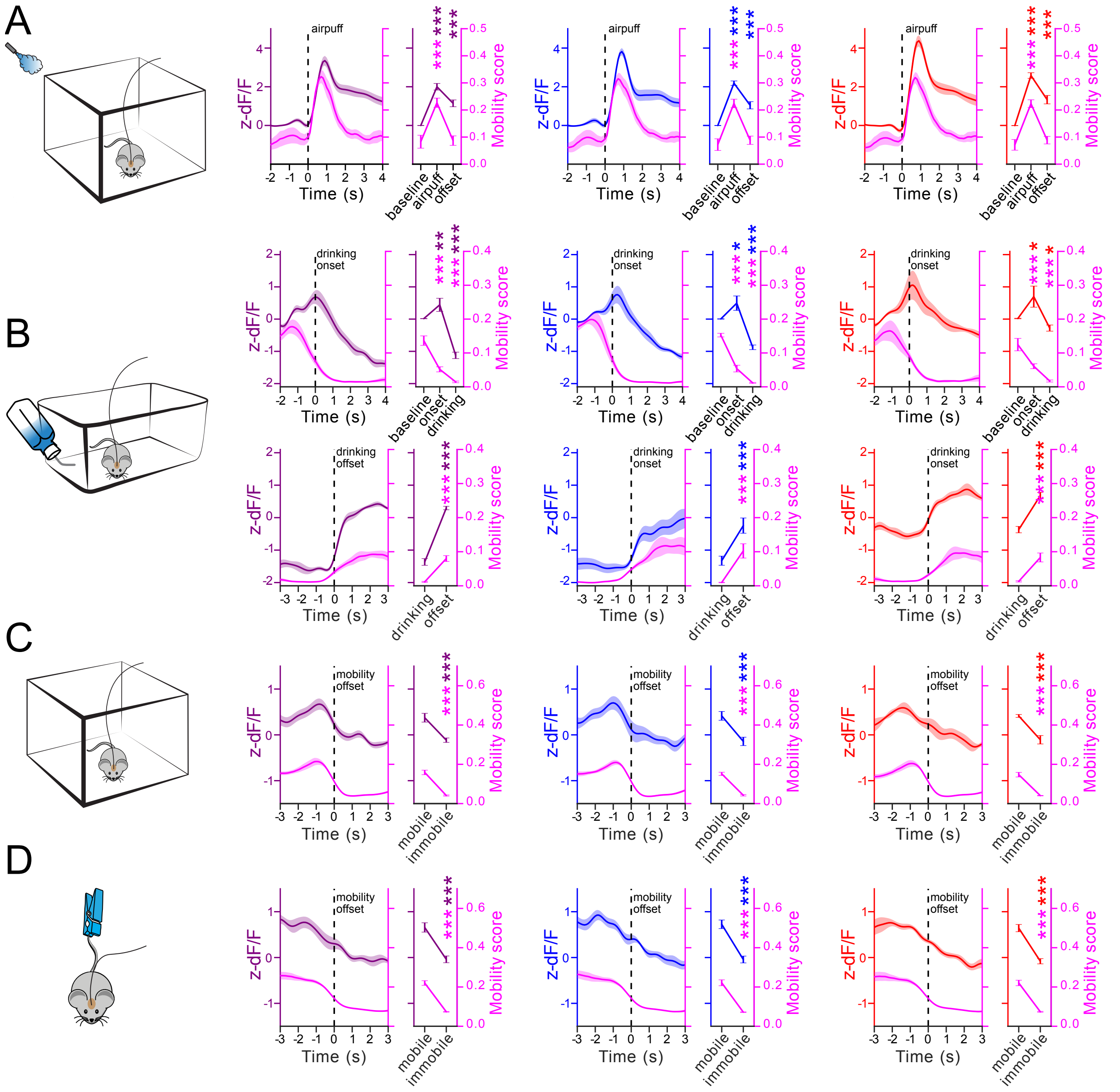

Figure S2. APT and SCT with movement, mobility offsets in OFT and TST. Related to Figure 2. (**A**) Peri-event plot of the average Ca^{2+} signal traces with all airpuff events at the LH→DRN, LH→VTA, and LH→LHb axon terminals and mobility score (magenta line). Plot of area under the curve (AUC) before and after the airpuffs. The lines represent means ± SEM. Same convention as with B for the SCT (**B**) at drinking onset (top) and offset (bottom), the OFT (**C**) and the TST (**D**) at mobility offset. Repeated measures two-way ANOVA within factors pathway (LH→DRN, LH→VTA, and LH→LHb), and time period (different for each test) with post hoc Dunnett's test. The p values were adjusted using the Bonferroni multiple testing correction method. In each test, around 5-10 mice were used and around 5-30 events (airpuffs, sucrose consumptions, mobility onsets) per mouse were produced. *p < 0.05, **p < 0.01, ***p < 0.001.

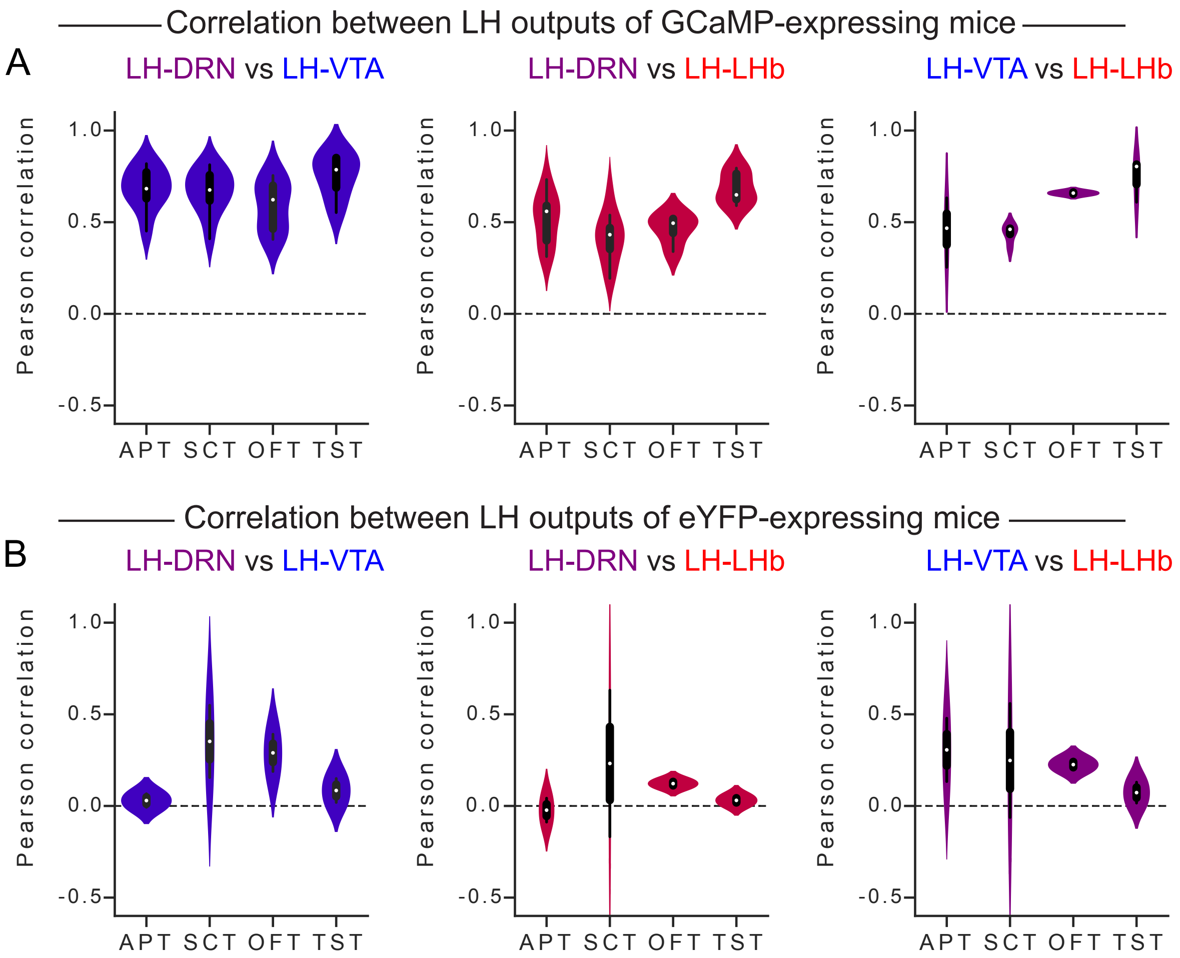

Figure S3. Pearson correlation between the signals recorded at the LH→DRN, LH→VTA, and LH→LHb pathways in mice expressing GCaMP6s and eYFP. Related to Figure 2. (**A**) Pearson correlation between the Ca^{2+} signals recorded at the LH→DRN, LH→VTA, and LH→LHb pathways in mice expressing GCaMP6s. (**B**) Pearson correlation between the Ca^{2+} signals recorded at the LH→DRN, LH→VTA, and LH→LHb pathways in mice expressing eYFP. Three-way ANOVA between factors group (GCaMP6s- and eYFP-expressing mice), and within factors pathway (LH→DRN, LH→VTA, and LH→LHb), and tests (APT, airpuff test; SCT, sucrose consumption test; OFT, open field test; TST, tail suspension test) with post hoc Tukey test. The p values were adjusted using the Bonferroni multiple testing correction method. The main effect was the difference between the mice expressing GCaMP6s and eYFP ($p < 0.05$). One sample t-test showed significant difference from 0 in mice expressing GCaMP6s ($p < 0.05$), but not in mice expressing eYFP ($p > 0.2$).

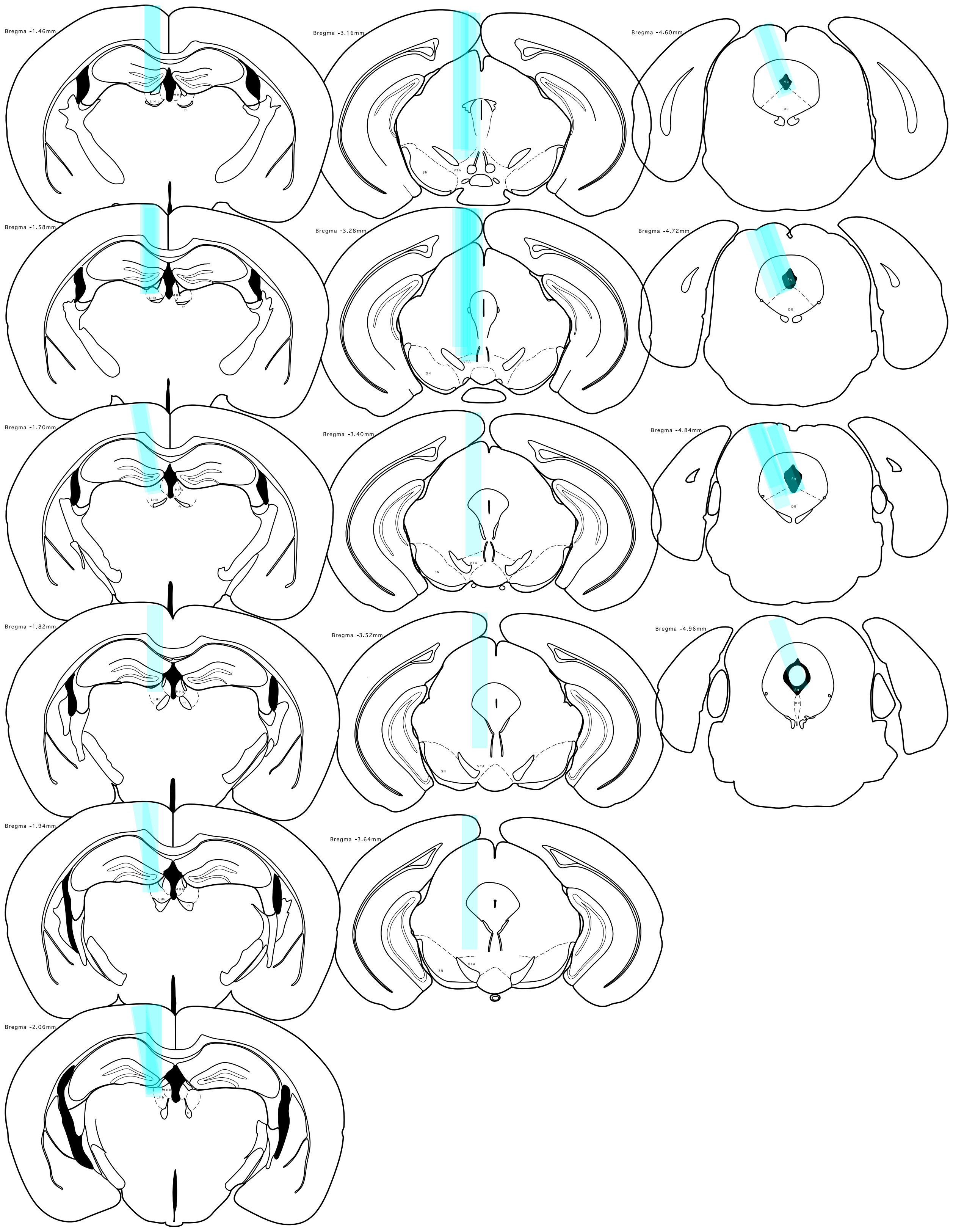

Figure S4. Cannulae placement in mice expressing GCaMP6s in the LHb (left), the VTA (middle), and the DRN (right). Related to Figure 2.

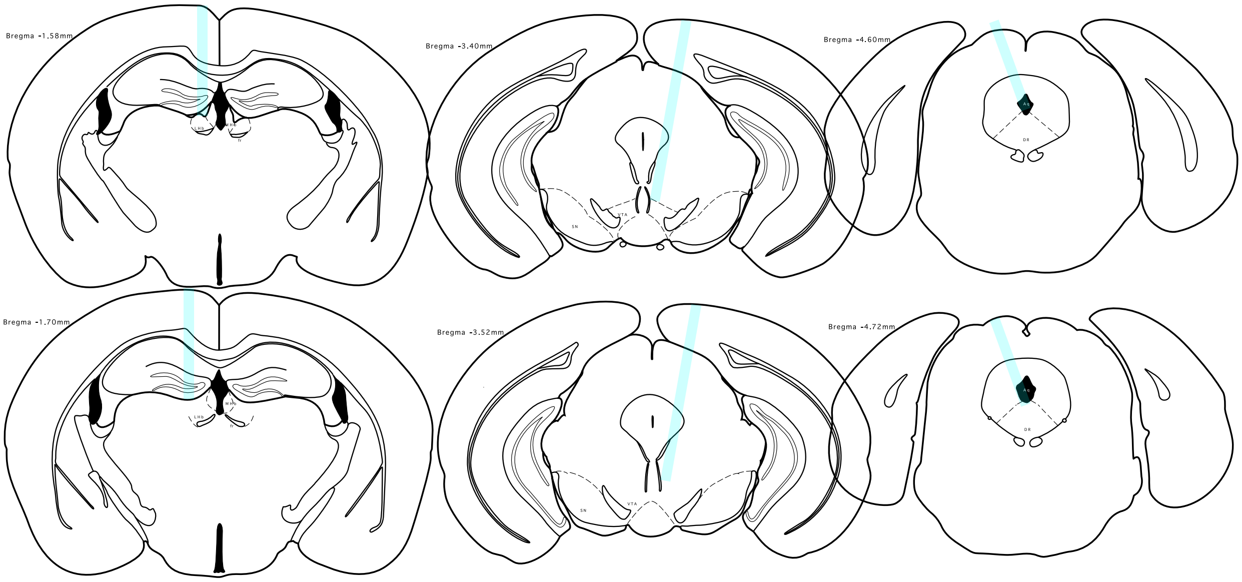

Figure S5. Cannulae placement in the mice expressing eYFP in the LHb (left), the VTA (middle), and the DRN (right). Related to Figure 2.

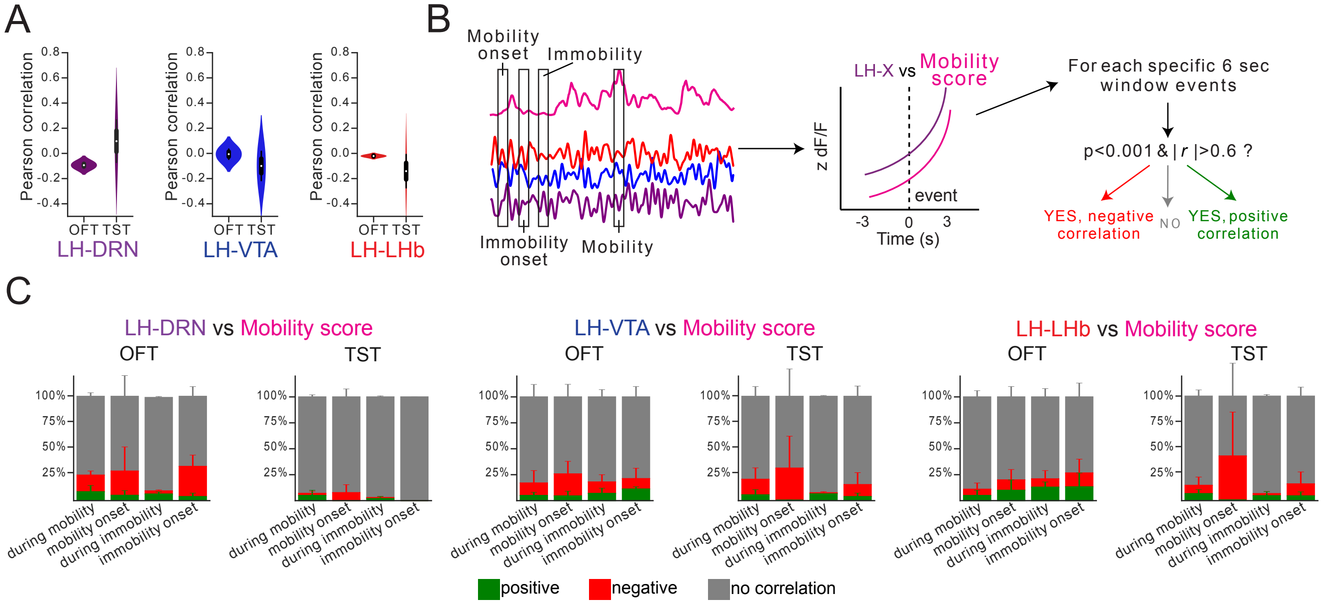

Figure S6. Correlation analysis between the signal at one of the LH neuronal output pathways and mobility score in mice expressing eYFP. Related to Figure 3. (**A**) The Pearson correlation between the Ca^{2+} signal measured at the LH→DRN, LH→VTA, and LH→LHb pathways and the mobility score during a complete session in the OFT or the TST. (**B**) Schematic of the event selection. Events at the onset of mobility and immobility and random events during mobility and immobility were chosen, and the Pearson correlation at 6-seconds peri-events between the Ca^{2+} signal and the mobility score was calculated. Correlations with $p < 0.001$ and $r > 0.6$ were considered as positive, $p < 0.001$ and $r < 0.6$ as negative, the others as not correlated. (**C**) Fraction of positive (green), negative (red), and uncorrelated events (gray) in the OFT and TST for the LH→DRN, LH→VTA, and LH→LHb pathways. Statistical analysis was performed along with the data from the mice expressing GCaMP6s.

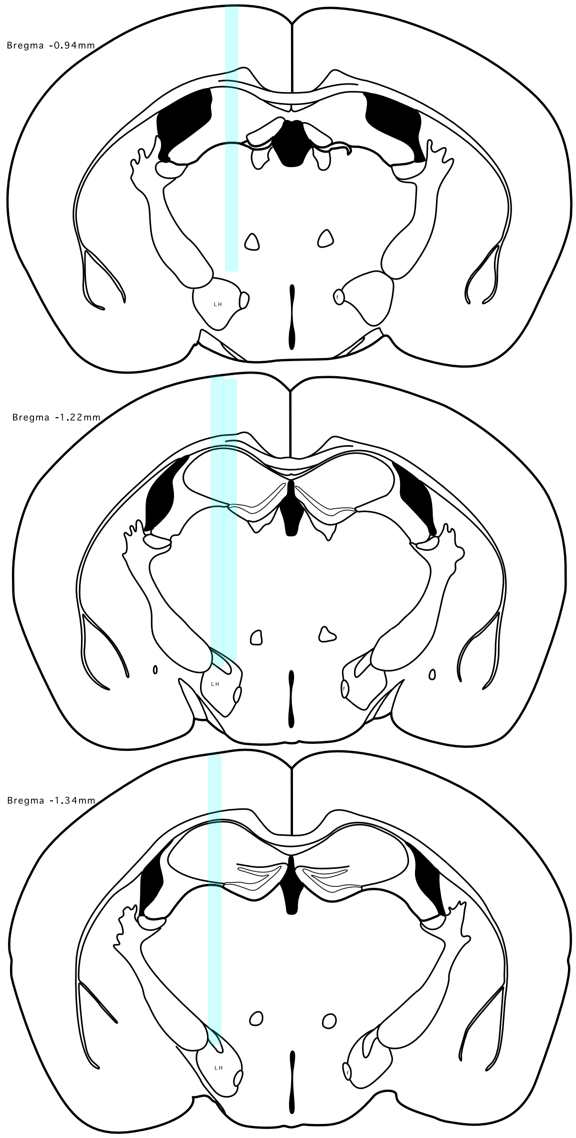

Figure S7. The cannulae placement in mice prepared using the intersectional viral strategy. Related to Figure 4.

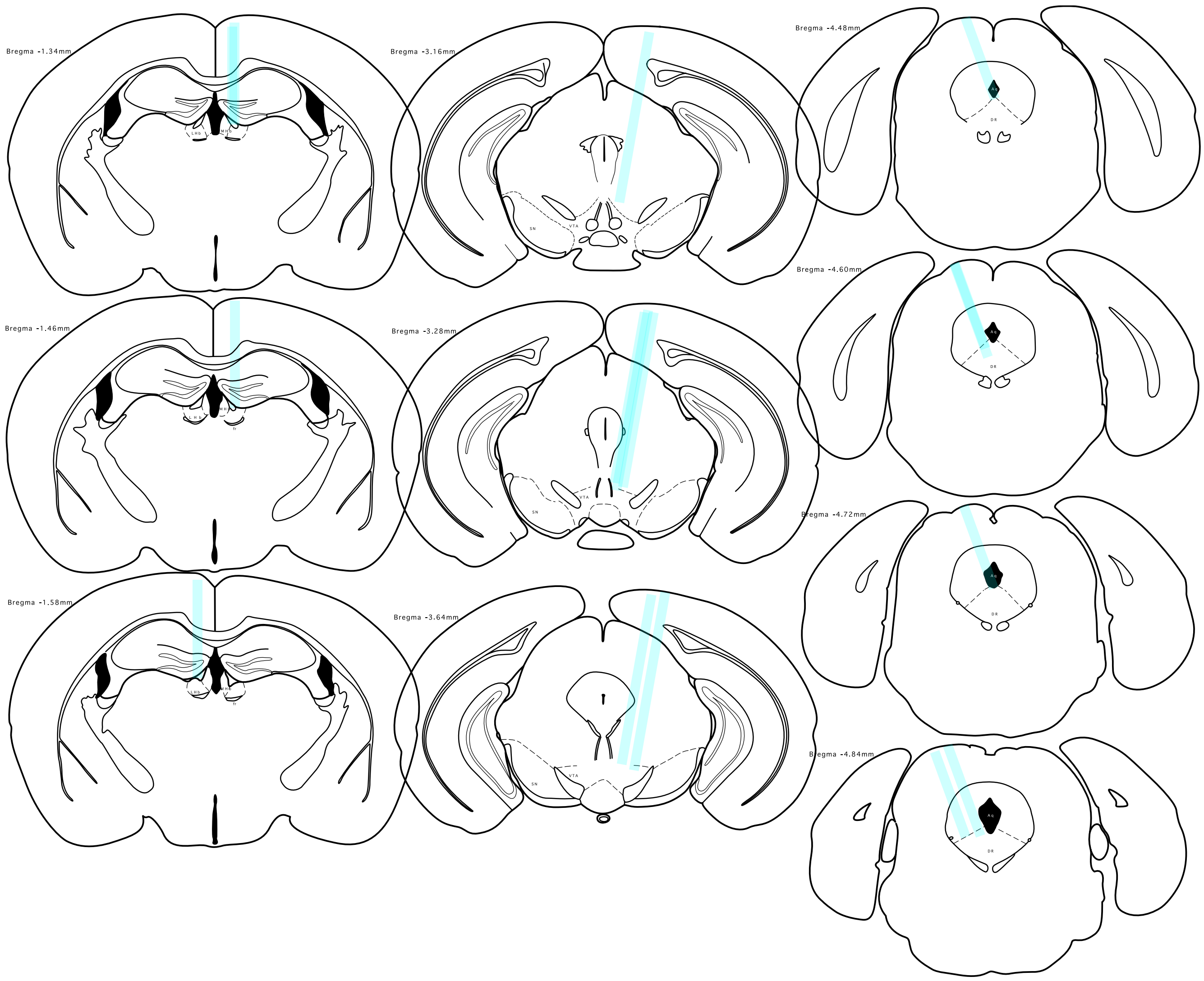

Figure S8. Cannulae placement in the LHb (left), the VTA (middle), and the DRN (right) of mice tested in the active avoidance task. Related to Figure 5.

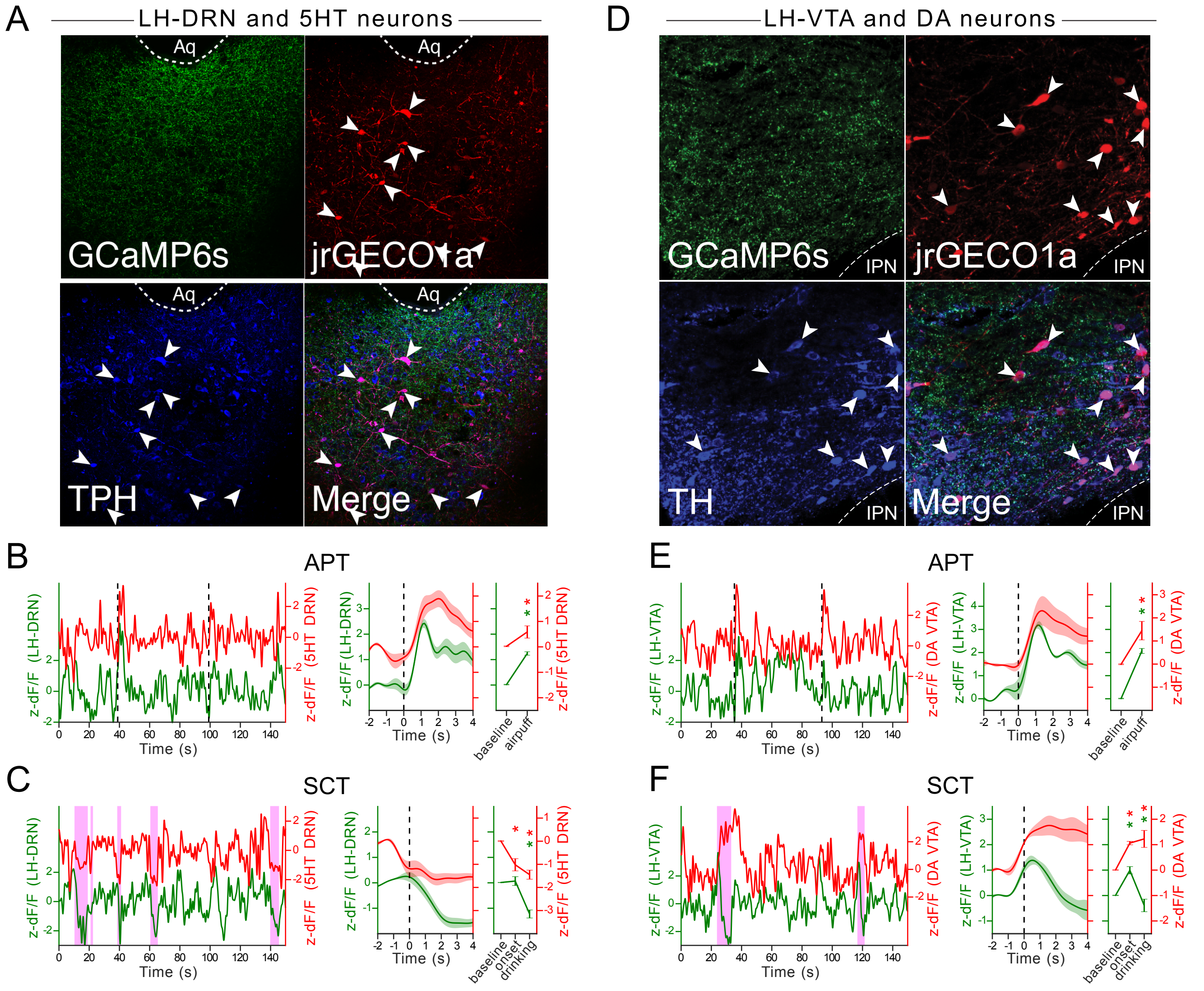

Figure S9. Simultaneous recordings at the DRN^{5HT} neurons and the LH→DRN pathway, and at the VTA^{DA} neurons and the LH→VTA pathway in the AP and the SCT. Related to Figure 7. (**A, D**) Confocal images of the DRN neurons (**A**) and VTA neurons (**D**) expressing jrGECO1a and immunolabelled for the serotoninergic marker tryptophane hydroxylase (TPH, blue **A**) or the dopaminergic marker tyrosine hydroxylase (TH, blue **D**). Shown in green are the LH axon terminals in the DRN and VTA that are expressing GCaMP6s. Representative Ca^{2+} signal traces recorded from the DRN^{5HT} neurons and at the LH→DRN pathway (**B**) or from the VTA^{DA} neurons and at the LH→VTA pathway (**E**) in mice presented with airpuffs (**B, E**) (left). Peri-event plots of the average Ca^{2+} signal traces with all the onset of mobility and the plots for the AUC at baseline and after the airpuffs (right). The lines represent means ± SEM. Same convention as for B, E for the SCT (**C, F**). Repeated measures two-way ANOVA within factors pathways (DRN^{5HT} and LH→DRN or VTA^{DA} and LH→VTA) and time periods (during immobility and mobility, at mobility pre-onset and onset) with post hoc Dunnett's test. The p values were adjusted using the Bonferroni multiple testing correction method. *$p < 0.05$, **$p < 0.01$, ***$p < 0.001$.

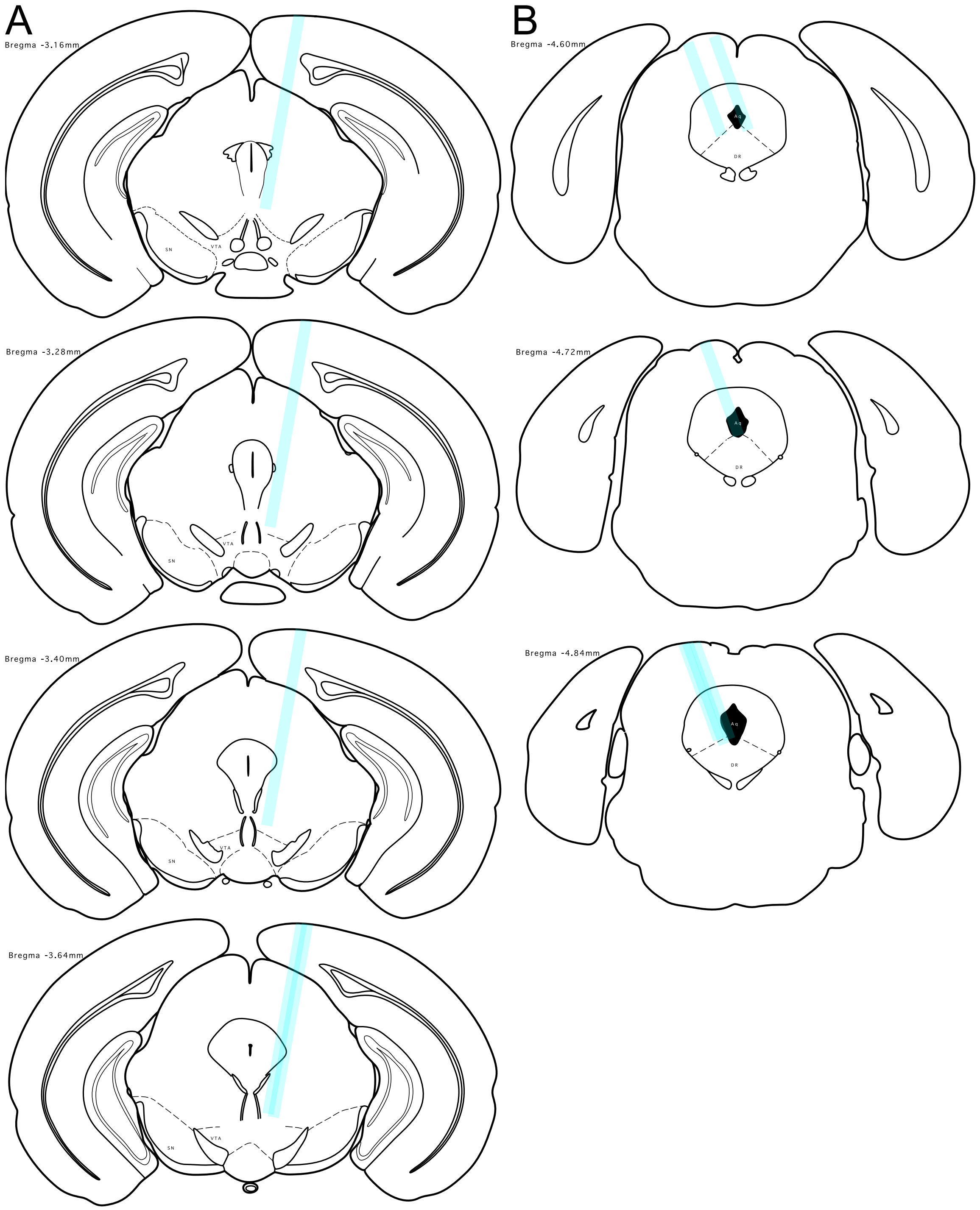

Figure S10. Cannulae placement in the ePet-cre (A) and DAT-ires-cre (B) mice. Related to Figure 7.

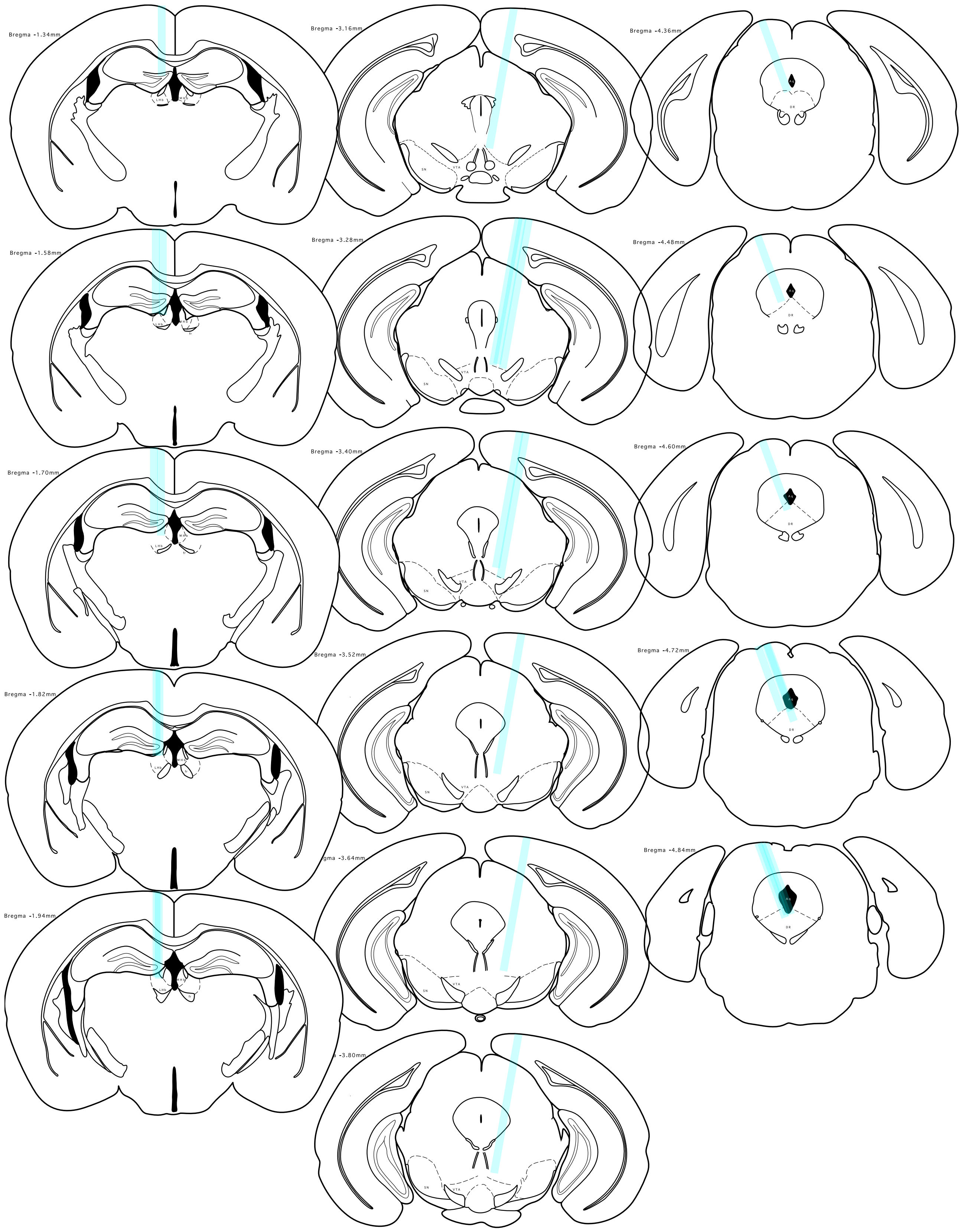

Figure S11. Cannulae placement in the LHb (left), the VTA (middle), and the DRN (right)in mice expressing ChR2-eYFP. Related to Figure 8.

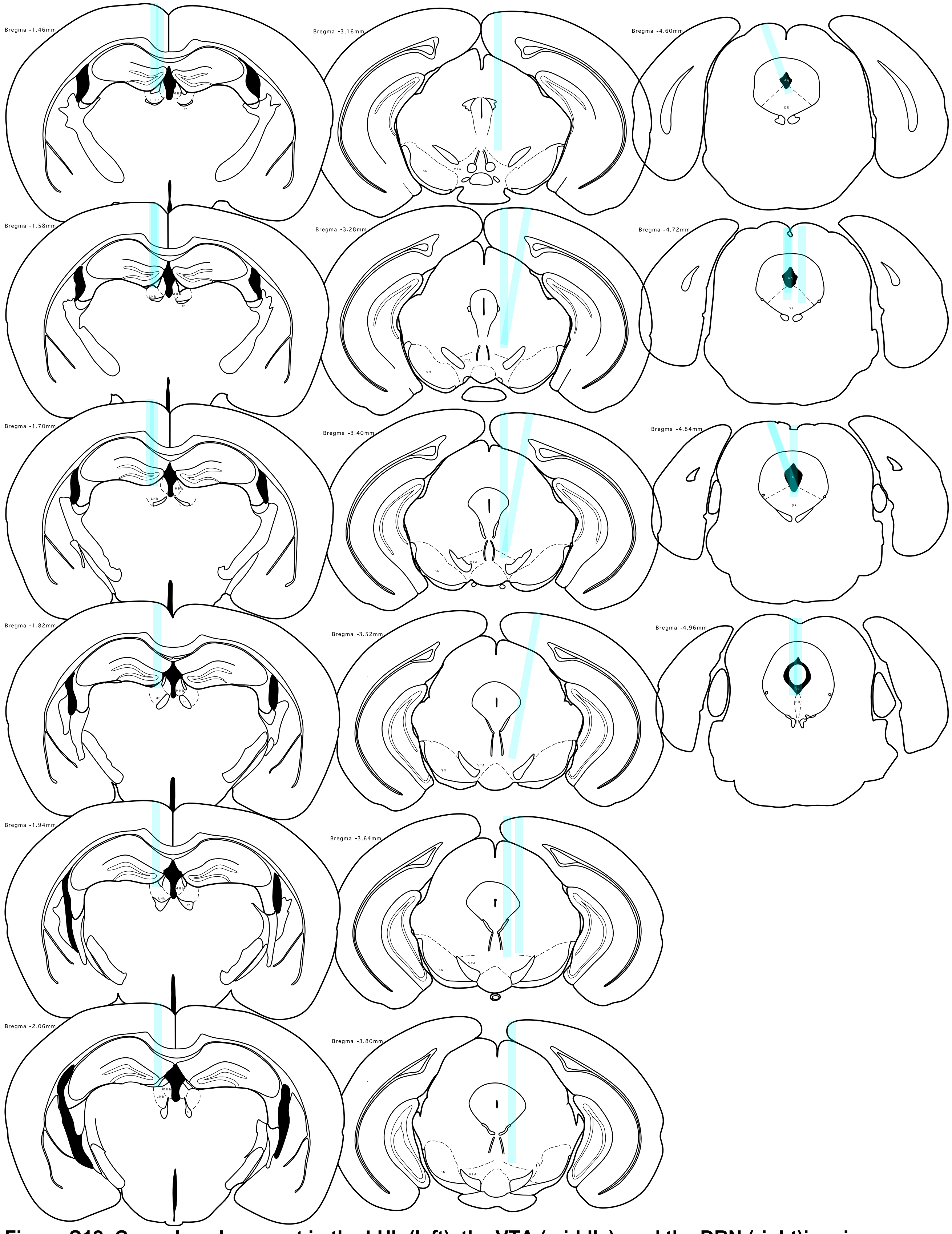

Figure S12. Cannulae placement in the LHb (left), the VTA (middle), and the DRN (right)in mice expressing eYFP. Related to Figure 8.
